# Electrochemical detection of anti-tissue transglutaminase antibody using quantum dots-doped polypyrrole-modified electrode

**DOI:** 10.1007/s00604-024-06620-w

**Published:** 2024-08-17

**Authors:** Cristina Dumitriu, Andreea Madalina Pandele, Mihaela Vasilica Mîndroiu, Oana-Andreea Lazar, Alina Popp, Marius Enachescu, George-Octavian Buica

**Affiliations:** 1 National University of Science and Technology Politehnica Bucharest, 313 Splaiul Independentei, Sector 6, 060042 Bucharest, Romania; 2 National Institute for Mother and Child Health “Alessandrescu-Rusescu”, 120 Lacul Tei Boulevard, Sector 2, 020395 Bucharest, Romania; 3 Center for Surface Science and Nanotechnology, National University of Science and Technology Politehnica Bucharest, 313 Splaiul Independentei, Sector 6, 060042 Bucharest, Romania; 4 Academy of Romanian Scientists, Splaiul Independentei 54, 050094 Bucharest, Romania

**Keywords:** Polypyrrole-modified electrode, Carbon quantum dots, Electroanalysis, Differential pulse voltammetry, Modified glassy carbon electrode, Anti-tTG antibody detection

## Abstract

A nanohybrid-modified glassy carbon electrode based on conducting polypyrrole doped with carbon quantum dots (QDs) was developed and used for the electrochemical detection of anti-tissue transglutaminase (anti-tTG) antibodies. To improve the polypyrrole conductivity, carrier mobility, and carrier concentration, four types of carbon nanoparticles were tested. Furthermore, a polypyrrole-modified electrode doped with QDs was functionalized with a PAMAM dendrimer and transglutaminase 2 protein by cross-linking with *N*-hydroxysuccinimide (NHS)/*N*-(3-dimethylaminopropyl)-*N*′-ethylcarbodiimide hydrochloride (EDC). The steps of electrode surface modification were surveyed via electrochemical measurements (differential pulse voltammetry (DPV), impedance spectroscopy, and X-ray photoelectron spectroscopy (XPS)). The surface characteristics were observed by scanning electron microscopy (SEM), Fourier transform infrared (FTIR) spectroscopy, and contact angle measurements. The obtained modified electrode exhibited good stability and repeatability. DPV between − 0.1 and 0.6 V (vs. Ag/AgCl 3 M KCl reference electrode) was used to evaluate the electrochemical alterations that occur after the antibody interacts with the antigen (transglutaminase 2 protein), for which the limit of detection was 0.79 U/mL. Without the use of a secondary label, (anti-tTG) antibodies may be detected at low concentrations because of these modified electrode features.

**Graphical Abstract:**

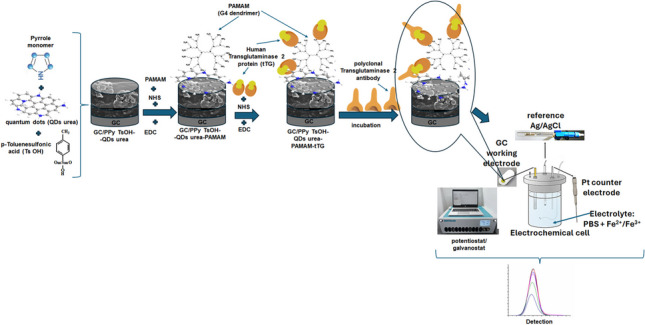

**Supplementary Information:**

The online version contains supplementary material available at 10.1007/s00604-024-06620-w.

## Introduction

The prevalent gastrointestinal ailment coeliac disease (CD) is thought to affect up to 1% of people worldwide [1]. While the biopsy-based diagnosis of coeliac disease is considered the gold standard, it is not without its drawbacks [2]. These considerations led to the first adoption of a serology-based diagnosis in the European Society for the Study of Pediatric Gastroenterology, Hepatology, and Nutrition (ESPGHAN) guidelines [2]. Mucosal atrophy occurs when the level of anti-tissue transglutaminase (anti-tTG) antibodies is at least 10 times the upper limit of the normal range (1 U/mL). Anti-tTG antibody levels have a strong positive predictive value (PPV) for CD diagnosis and may also be used to monitor this disease [3].

When diagnosing CD clinically, the most common serological test is an enzyme-linked immunosorbent assay (ELISA) with optical detection [4]. In ELISA, antibodies are identified by several steps (incubation, washing, and separation procedures) and require complex equipment [5, 6]. Therefore, quicker, more accurate, cheaper, and simpler point-of-care devices need to be developed. Electrochemical sensors are known for being very sensitive, selective, flexible, and easy to use [7] and for having smaller sample sizes, which saves money [8]. Other attributes include suitable electroanalytical qualities, simplicity and affordability, design versatility, small dimensions, and the potential for inclusion in portable systems [9]. Recently, different surface chemistry methods, mainly anti-gliadin and anti-tTG, have been used to detect autoantibodies specific to CD [3–9].

More recent efforts to enhance the sensitivity and specificity of electrochemical sensors have relied on techniques based on nanomaterials [5]. The conductive polymer polypyrrole (PPy) has several important characteristics: it is biocompatible (both in vitro and in vivo) and can enhance the durability and electrocatalytic activity of the electrode surface [10]. The electrode’s electrochemical performance may be influenced by conductivity; increasing PPy’s conductivity can be achieved by doping target polymer chains with foreign anions (p-type doping) or cations (n-type doping) to lower the energy barrier and facilitate electron movement. For instance, *p*-toluenesulfonic acid, also known as tosylic acid (TsOH), was used as a dopant because it dissolves in water and can change the shape and structure of the conducting polymer [11]. When TsOH is added, the conductivity, carrier mobility, and carrier concentration are improved compared with those of inorganic modified conducting polymers [11].

The interest of the international scientific community in carbon quantum dots (QDs) or graphene quantum dots has grown in recent decades. These materials have multiple reaction sites due to their high surface-to-volume ratios. Charge and intermediate transfers are limited in QDs due to their quantum size and intermediate nature [12]. The hydrophilic groups on their surfaces (hydroxyl, carboxyl, etc.) make them easy to functionalize, boosting their catalytic influence on redox processes [13]. Also, due to their stellar reputation as electron donors and acceptors, GQDs are fascinating materials for electrode production [14]. GQD/PPy hybrids provide excellent specific capacitance, rate capability, and cycling stability due to the mechanical robustness of QDs [15]. Compared to densely structured plain PPy, QD-doped PPy has a porous structure, more active edges, and more conductive routes for increasing the catalytic current density and decreasing the charge transfer resistance [16]. Poly(amidoamine) (PAMAM) dendrimers are used in electrochemical biosensors because their branching (tree-like) polymeric structures include several amino groups for conjugation with carboxylated probes. Thus, the remarkable qualities of GQDs and PAMAM may be combined and studied to produce a diagnostic tool that detects and quantifies target analytes [5].

In the present work, electrochemical detection of anti-tTG antibody levels was performed with a modified GC electrode. The electrode was designed starting with four types of carbon nanoparticle preparation. The ones showing the highest signal in DPV were selected as dopants for polypyrrole. To be used for transglutaminase 2 antibody level detection, a PAMAM dendrimer and transglutaminase 2 protein (tTG) were grafted onto a selected doped polypyrrole film. The new polypyrrole-QD-PAMAM-tTG-modified electrode based on the antigen–antibody reaction was electrochemically monitored by differential pulse voltammetry (DPV). Even though the proposed method requires a complex nanomaterial to be prepared, it can be easily prepared starting from inexpensive raw materials such as citric acid, urea, and pyrrole. These modified electrodes are intended to be used for very fast results in CD diagnosis and monitoring, even at low antibody levels, using a small amount of sample and without the need for a secondary label.

## Experimental

### Reagents

*p*-Toluenesulfonic acid monohydrate (97%) and folic acid dihydrate (≥ 97%) were obtained from Thermo Fisher Scientific (USA); citric acid (ACS reagent, ≥ 99.5%), sodium hydroxide (reagent grade, 97%), L-cysteine (97%), urea (ACS reagent, 99.0–100.5%), PAMAM dendrimer (ethylenediamine core, generation 4.0 solution 10 wt.% in methanol), *N*-(3-dimethylaminopropyl)-*N*′-ethylcarbodiimide hydrochloride (EDC) (commercial grade, powder), *N*-hydroxy succinimide (NHS) (98%), potassium ferricyanide [K_3_Fe(CN_6_)] (99%), potassium ferrocyanide K_4_[Fe(CN)_6_] (ACS reagent, 98.5–102.0%), sodium phosphate monobasic (NaH_2_PO_4_) (anhydrous, ≥ 99%), sodium phosphate dibasic (Na_2_HPO_4_) (puris, 98–100%), ethylene glycol (EG), and dimethyl sulfoxide (DMSO) (ACS reagent, ≥ 99.9%) were purchased from Sigma-Aldrich, USA; rabbit polyclonal transglutaminase 2 antibody 1 mg/mL and recombinant human transglutaminase 2 protein (tTG) were purchased from Abcam Limited UK. All used reagents were of analytical grade, and the solutions were prepared in Milli-Q purified water.

An enzyme-linked immunosorbent assay (ORGENTEC Diagnostika GmbH, Mainz, Germany) control ( −) and calibrators were used for the quantitative determination of the IgA and anti-tTG antibody concentrations.

### Equipment and methods

The supplementary material provides details about the equipment used and the analysis parameters.

### Carbon quantum dots (QDs) synthesis

Four different types of carbon quantum dots—QD citric, QD folic, QD Cys, and QD urea—were prepared. The synthesis and characterization of the QDs are provided in the supplementary material.

### Electrode modification with polypyrrole (PPy) and PPy/QDs films

Prior to surface modification, the GC electrodes were polished with Al_2_O_3_ powder using a Metrohm polishing kit and rinsed with distilled water. The second polishing step was performed with diamond paste (0.25 µm) on a polishing cloth (from Presi) until a mirror was finished, followed by rinsing with distilled water and allowing the mixture to dry in the air.

The GC electrodes were modified by controlled potential electrolysis at + 0.8 V vs. Ag/AgAgCl and 3 M KCl using a 1-mC polymerization charge under the following conditions:

- Electrode GC/PPy TsOH from a 0.2 M pyrrole monomer aqueous solution with a 0.1 M TsOH electrolyte.
- GC/PPy TsOH-QDs urea was prepared from a 0.2 M pyrrole monomer aqueous solution with 0.1 M TsOH added to each case: 7.5 mL QD solution/25 mL electrolyte solution.

### Electrode modification with PAMAM dendrimer and tTG protein

An aqueous solution containing 10 µL of PAMAM dendrimer/1 mL of solution with 0.4 M EDC and 0.1 M NHS was prepared. After mixing well with a vortex mixer, 30 µL of this solution was deposited on the GC/PPy TsOH-QDs urea electrode surface and allowed to react at room temperature for 1 h, resulting in a GC/PPy TsOH-QDs urea-PAMAM electrode.

To modify the electrode with the tTG protein, the first step involved dissolving 10 µg of recombinant human transglutaminase 2 protein (tTG) in 500 µL of 0.1 M PBS (pH 7.4; named solution 1). Second, an aqueous solution of 0.2 M EDC and 52 mM NHS was prepared (named solution 2). Finally, 10 µL of solution 1 and 20 µL of solution 2 were mixed well, deposited on a GC/PPy TsOH-QDs urea-PAMAM electrode, and incubated for 3 h at room temperature to facilitate the establishment of amide bond interactions between the − COOH groups of tTG and the − NH_2_ groups of PAMAM. The unbound antigen (tTG) was eliminated after washing with 0.1 M PBS (pH 7.4). The GC/PPy TsOH-QDs urea-PAMAM-tTG electrode was allowed to dry at room temperature before use. Additionally, some electrodes were prepared and stored at 4 °C in a refrigerator for different measurements.

### Detection of anti-tTG antibodies

Assays were conducted using four freshly prepared GC/PPy TsOH-QDs urea-PAMAM-tTG electrodes incubated for 60 min in 10^−4^ mg/mL commercial rabbit polyclonal transglutaminase 2 antibody. Next, PBS (pH 7.4) was used to wash away the unbound tTG antigen solution. Then, DPV was conducted in a solution of 0.1 M PBS (pH 7.4) with 5 mM K_3_Fe(CN_6_) and 5 mM K_4_[Fe(CN)_6_]. The response of the GC/PPy TsOH-QDs urea-PAMAM-tTG electrode was further examined in the presence of commercial anti-tTG antibodies (Fig. 7b) between − 0.1 and 0.6 V (vs. Ag/AgCl, 3 M KCl reference electrode).

For the calibration curve, the electrodes were immersed in 30 µL of various antibody concentrations from calibrator reference solutions (ORGENTEC Diagnostika GmbH, Mainz, Germany) for a duration of 60 min. This was followed by washing with PBS (pH 7.4), and subsequent electrochemical DPV measurement was performed in 0.1 M PBS (pH 7.4) with a [Fe(CN)_6_]^3−/4−^ redox probe. Measurements were made in triplicate, and the average value was used.

### Ethical considerations

This research did not involve human or animal samples.

## Results and discussion

**Fig. 1:**
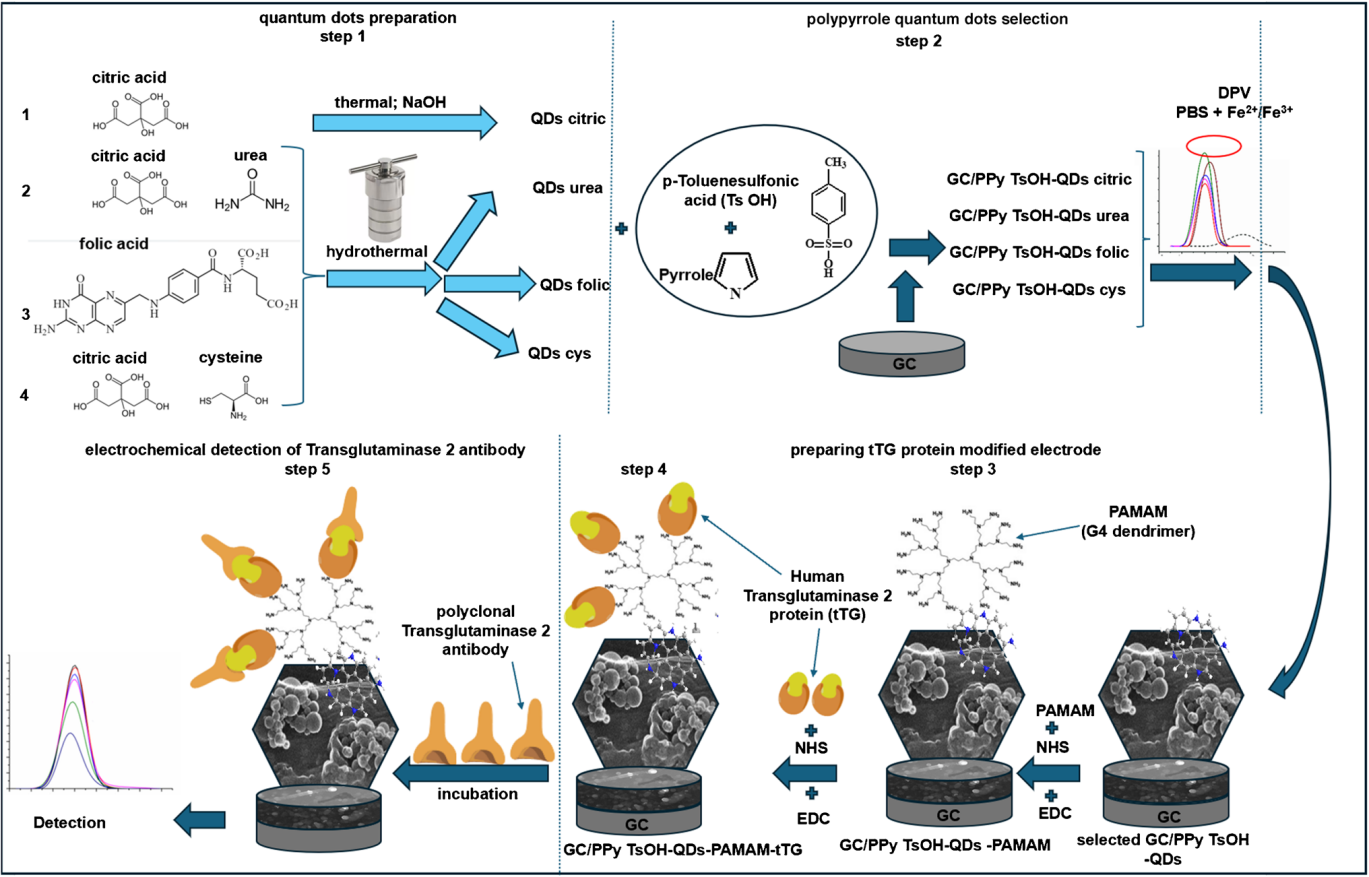
Process of developing a GC/PPy QDs-modified electrode for transglutaminase antibody electrochemical detection

Electrochemical detection of anti-tTG antibodies with modified electrodes was performed according to the schematic illustration in Fig. 1. During the synthesis phase, it is feasible to manipulate the inherent attributes of QDs by incorporating heteroatoms like sulfur (S) or nitrogen (N), either singularly or in distinct combinations. The first step (Fig. 1, step 1) consisted of the synthesis of four types of quantum dots (three of which are mentioned in the supplementary material). All the samples were spectroscopically characterized by FT-IR, UV, and fluorescence (Fig. S1 from the supplementary material). Comprehensive spectroscopic analyses involving FT-IR, UV, and fluorescence assessments were conducted on all samples (refer to Fig. S1 in the supplementary material). Subsequent to these evaluations, electrochemical tests were carried out to discern the QDs capable of enhancing the conductivity, carrier mobility, and carrier concentration of the conducting polymer polypyrrole (as depicted in Fig. 1, step 2). Notably, the electrochemical assessments revealed a significant augmentation in current levels across all QDs in comparison to GC/PPy TsOH when subjected to a DPV test utilizing 0.1 M PBS at pH 7.4, 5 mM K3[Fe(CN)6], and 5 mM K4[Fe(CN)6]. Among these, the GC/PPy TsOH-QDs urea electrode demonstrated the highest peak height (illustrated by the green line), with a remarkable 49% increase in the peak current when compared to GC/PPy TsOH. Upon examination of Fig. S2a from the supplementary materials, it is evident that all quantum dots (QDs) exhibit heightened currents in comparison to the GC/PPy TsOH when subjected to a differential pulse voltammetry (DPV) test using 0.1 M PBS at pH 7.4 along with 5 mM K_3_[Fe(CN)_6_] and 5 mM K_4_[Fe(CN)_6_]. Notably, the GC/PPy TsOH-QDs urea electrode displays the most prominent peak height (depicted by the green line), with a notable 49% increase in current over the GC/PPy TsOH configuration. Furthermore, based on the slope analysis from Fig. S2b, the band gap energy of the GC/PPy TsOH-QDs urea composite is lower compared to that of GC/PPy TsOH. Analysis of Fig. S2 c reveals a higher Urbach energy for the GC/PPy TsOH-QDs urea, indicating an elevated presence of defects resulting from polymerization within the monomer and TsOH-QDs urea solution. This suggests enhanced conductivity due to the heightened doping level of the polymer film under these circumstances. In conclusion, for the subsequent electrode modification phase, only QDs urea was chosen for experimentation, as both the PAMAM dendrimer and the tTG enzyme proved to be excessively costly for comprehensive testing.

The PAMAM dendrimer was subsequently tested due to its tree-like polymeric structure and several amino groups that can bind to probes that possess carboxylate functional groups. There are two methods available to link dendrimers to PPy-QDs film: cyclic voltammetry or carbodiimide-succinimide (NHS/EDC) cross-linking (Fig. 1, step 3). Modified electrodes were prepared using each of them. The NHS/EDC system was selected because the electrode gave rise to a higher peak current during DPV for the [Fe(CN)_6_]^3−/4−^ redox probe. Furthermore, PAMAM allowed the binding of the transglutaminase 2 protein for specific transglutaminase 2 antibody detection. The proteins were also linked using EDC/NHS chemistry (Fig. 1, step 4). It has been previously demonstrated [17] that the substantial surface coverage of PPy with PAMAM G4 confers numerous benefits. This includes facilitating the connection of aptamers for enhanced efficacy, broadening the dynamic range of prion protein detection, streamlining the fabrication process due to its simplicity and efficiency, and enabling direct measurement without the need for amplification. Furthermore, in the development of a sensor for *Mycobacterium* tuberculosis utilizing MWCNTs/PPy/PAMAM, a strategic approach was employed to prevent non-specific interactions between MWCNTs and DNA. This was achieved through the incorporation of MWCNTs within a PPy film and the addition of PAMAM G4 on the surface [18]. Building upon these advantages, our study aims to investigate the response of the modified electrode to varying concentrations of transglutaminase 2 antibodies specific to CD in the final step of our experimental procedure (Fig. 1, step 5).

### QDs urea and modified electrode surface characterization

**Fig. 2:**
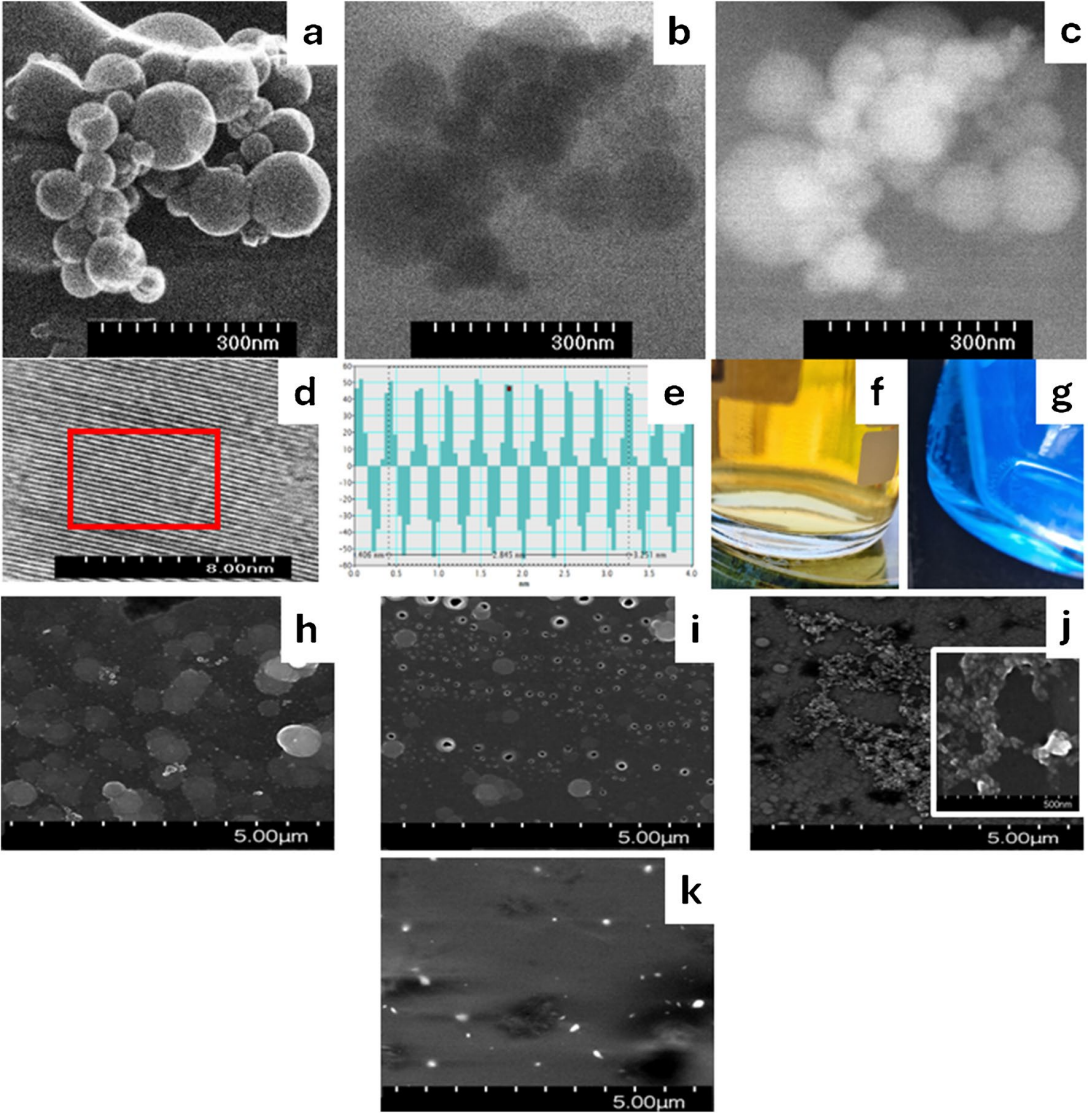
HR-STEM images of QDs urea sample acquired using different imaging modes: SE (**a**), ZC (**b**), and TE (**c**); high-resolution STEM image (**d**); corresponding profile of the red square from (**e**); optical images under the illumination of daylight (**f**) and UV (365 nm) light (**g**); and SEM images for modified electrodes GC/PPy TsOH (**h**), GC/PPy TsOH-QDs urea (**i**), GC/PPy TsOH-QDs urea-PAMAM (**j**), and GC/PPy TsOH-QDs urea-PAMAM-tTG (**k**)

To obtain more information on the selected QDs urea, scanning transmission electron microscopy (STEM) was used. The results are presented in Fig. 2. These images were obtained using different detectors: SE, secondary electrons (Fig. 2a); ZC, phase contrast (Fig. 2b); and TE, transmission electrons (Fig. 2c), at the same location on the sample at the same magnification (× 1000 K). A high-resolution STEM image presented in Fig. 2d was obtained using the bright-field detector/TEM image. The corresponding profile for the interatomic distance calculated through the TE image (marked in the red square) is presented in Fig. 2e which corresponds to the interplanar distance of the investigated sample was determined from Fig. 2d. By using the ZC phase contrast, we were able to clearly observe QDs with spherical and nonuniform shapes and with different diameter sizes. The as-prepared QDs urea is typically agglomerated. Figure 2d is inserted into specific software to which a fast Fourier transform (FFT) function was applied. As a result, an FFT image was obtained, and a mask was applied by choosing two bright points. The inverse fast Fourier transform (IFFT) function was then applied to determine the appropriate profile for determining the interplanar distance value. As you can see in Fig. 2e, an average value of 2.845 nm appears, but this value is divided among the 8 parts and the final *d*-spacing value of 0.3556 nm was obtained, which may correspond to the (002) crystal phase of graphite [19].

The aqueous solution of the urea QDs was yellow in daylight (Fig. 2f) and exhibited blue fluorescence when exposed to 365-nm UV light (Fig. 2g).

Scanning electron microscopy (SEM) was used to obtain detailed morphological changes after each stage of the modification, and the images are shown in Fig. 2h, i, j, and k. In Fig. 2h, the electro-polymerization process resulted in the formation of a homogeneous and porous PPy TsOH layer with hierarchical, upwardly oriented nanostructures, different from the classical cauliflower-like morphology. It has previously been shown [20] that pyrrole monomers undergo electrochemical oxidation on a glassy carbon surface, which results in oligomers. Subsequently, the use of a “soft” template consisting of TsOH anions facilitates the vertical aggregation of PPy conglomerates at the nucleation sites, resulting in the formation of one-dimensional nanostructures that can facilitate efficient electron transport and ion diffusion. In Fig. 2i, in the case of the GC/PPy TsOH-QDs urea nanostructured film**,** no significant morphological alterations are apparent compared to those of the GC/PPy TsOH. By analyzing the functional groups identified in the FT-IR spectra, contact angle, and surface energy values, it was observed that the GC/PPy TsOH-QDs urea film exhibited a decrease in contact angle (indicating increased hydrophilicity) and an elevation in surface energy, with a water contact angle of 56°, in contrast to the GC/PPy TsOH film with a water contact angle of 82°. This suggests that the incorporation of QDs in the PPy film introduced additional hydroxyl (OH) and carboxyl (COOH) groups, thereby enhancing its hydrophilic properties and facilitating the further binding of PAMAM dendrimer. In the inset of Fig. 2j, the presence of a small globular polymeric structure on the GC/PPy TsOH-QDs urea-PAMAM electrode surface confirms the attachment of the dendrimer PAMAM. As shown in Fig. 2k, for the GC/PPy TsOH-QDs urea-PAMAM-tTG electrode surface, the small globular polymeric structure of PAMAM was no longer visible and was probably covered by the tTG enzyme.

Elemental mapping was conducted to evaluate the consistency of the samples and verify the dispersion of carbon (C), nitrogen (N), and sulfur (S) after each stage of electrode modification. The results of this analysis are shown in Fig. S3 in the supplementary material. All the PPy, QDs, PAMAM, and tTG protein contained C and N. The *p*-toluenesulfonic acid and tTG protein (with cysteine residues) contained sulfur. The distributions of C, N, and S in the films are uniform for the GC/PPy TsOH (Fig. S3a, b, c), GC/PPy TsOH-QDs urea (Fig. S3d, e, f), and GC/PPy TsOH-QDs urea-PAMAM samples (Fig. S3 g, h, i). The GC/PPy TsOH-QDs urea-PAMAM-tTG sample surface (Fig. S3 j, k, l) has some agglomerations. The three elements C, N, and S were detected at high intensities in all the samples, and they were present with equal distributions.

**Fig. 3:**
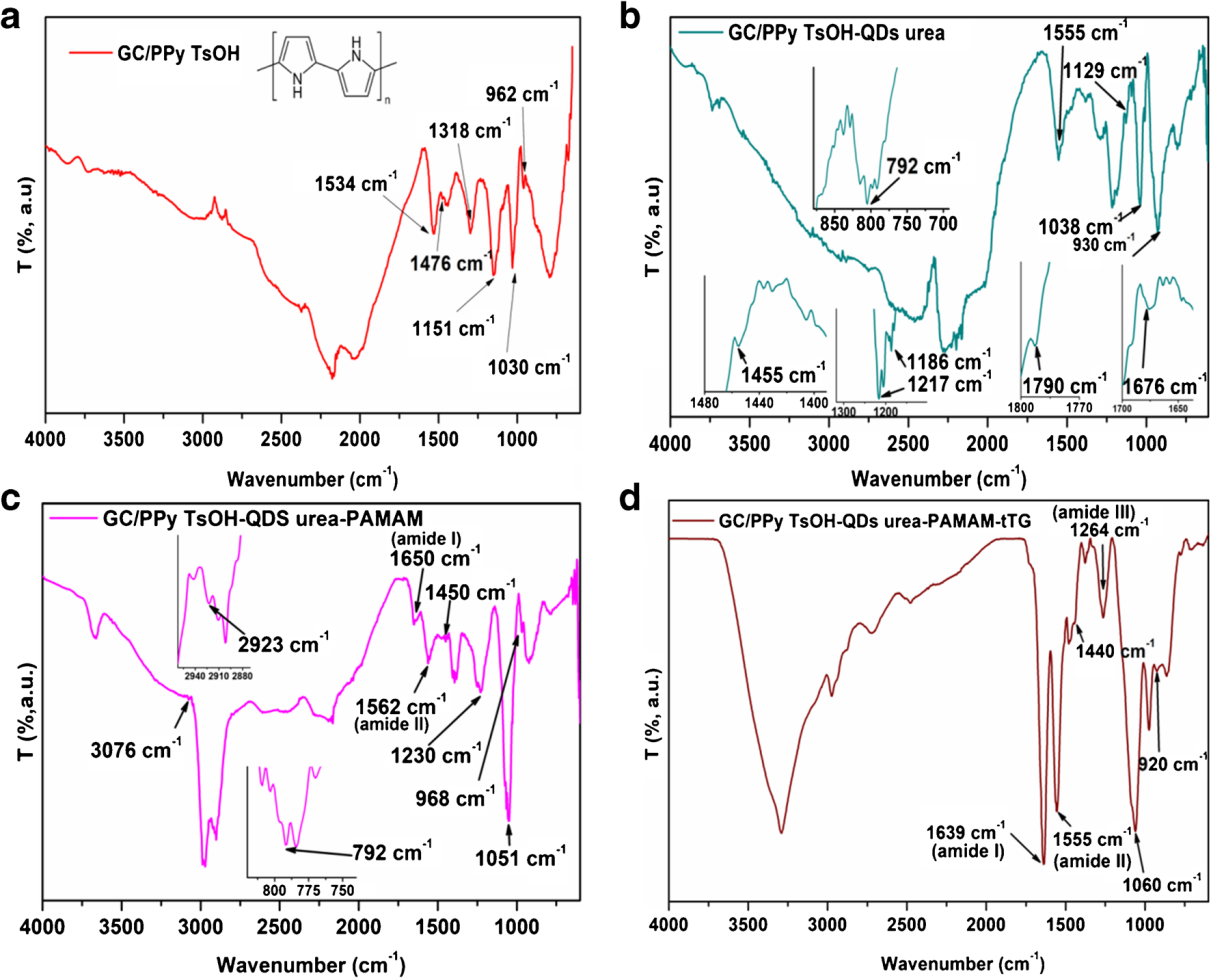
The FT-IR spectra for GC/PPy TsOH (**a**), GC/PPy TsOH-QDs urea (**b**), GC/PPy TsOH-QDs urea-PAMAM (**c**), and GC/PPy TsOH-QDs urea-PAMAM-tTG (**d**)

The FT-IR spectra of the GC/PPy TsOH-modified electrode are presented in Fig. 3a. The stretching vibration of the C = C and C–N bonds in the pyrrole ring is associated with the noticeable peaks at 1534 and 1476 cm^−1^ in the infrared spectra [21]. The presence of TsOH is shown by the characteristic antisymmetric and symmetric stretching vibration peaks of O = S = O, which can be observed at 1151 and 1030 cm^−1^, respectively [21]. The deformations of the C–H, O–H, and C–N groups are responsible for the peak at 1318 cm^−1^ [22]. The peak at 962 cm^−1^ can be attributed to the C–C deformation [23].

In Fig. 3b, we can observe the FT-IR spectra of the GC/PPy TsOH-QDs urea-modified electrode. The peak observed at 1790 cm^−1^ is related to the peak in the FT-IR spectrum of QDs urea. The fundamental vibration in the pyrrole ring may be traced to the bands at 1690 cm^−1^ and 1555 cm^−1^ [14]. The peak at 1555 cm^−1^ can also be caused by carboxylate COO^−^ asymmetric stretching [24]. The peak at 1455 cm^−1^ can be associated with the saturated C–H bending vibration or C–N bonds in the pyrrole ring [21, 25]. The vibrations caused by the stretching of C–N bonds account for the 1217 cm^−1^ band [22]. The stretching vibration of C–O caused a peak at approximately 1186 cm^−1^ [14]. It is possible that the C–N bonds are responsible for the peak at 1129 cm^−1^ [26]. The C–H bond in-plane vibration is responsible for the peak at 1038 cm^−1^ [14, 21]. The presence of a peak at 930 cm^−1^ may be ascribed to the deformation of C–C bonds [23]. The peak at 790 cm^−1^ corresponds to out-of-plane N–H bending [27].

Figure 3c displays the FT-IR spectra of the GC/PPy TsOH-QDs urea-PAMAM composite. It has been shown that fourth-generation poly(amidoamine) (G 4.0 PAMAM) dendrimers may significantly enhance the selectivity and sensitivity of electrochemical processes due to their globular structure and surface groups of primary amines [28]. The existence of terminal primary amino groups was indicated by the peak at 3076 cm^−1^ [29]. The distinctive amide I and II bands of PAMAM are observed at 1650 cm^−1^ and 1562 cm^−1^, respectively [28], implying that the G 4.0 dendrimer PAMAM was present on the modified electrode surface. There are also peaks in the FT-IR spectra observed and also in the GC/PPy TsOH/QDs urea spectra that are more or less shifted at 1450 cm^−1^, 1230 cm^−1^, 1051 cm^−1^, 968 cm^−1^, and 792 cm^−1^.

The FT-IR spectrum recorded for the GC/PPy TsOH-QDs urea-PAMAM-tTG-modified electrode is presented in Fig. 3d. The distinctive amide bands, visible in the FT-IR spectra of GC/PPy TsOH/QDs urea/PAMAM, are also observed in this spectrum and shift at wavenumbers of 1639 cm^−1^ and 1555 cm^−1^. The peak at approximately 1200 cm^−1^ is assigned in the literature to the stretching vibration of C–N bonds or amide III for a protein (related to N–H bending and C–H stretching vibrations) [22, 30]. Peaks observed in the spectra of GC/PPy TsOH/QDs urea and GC/PPy TsOH/QDs urea/PAMAM are also present in this sample spectrum at 1440 cm^−1^, 1060 cm^−1^, and 920 cm^−1^.

Contact angle measurements were made for every electrode modification stage, and the surface energy was calculated for the first two samples. Table S1 from the supplementary material highlights that every sample has a water contact angle of less than 90°, indicating its hydrophilicity. Based on the functional groups found in the FT-IR spectra, a decrease in the contact angle (increase in hydrophilicity) and an increase in the surface energy were obtained for the GC/PPy TsOH-QDs urea film (water contact angle 56°) in comparison to those of the GC/PPy TsOH film (water contact angle 82°), meaning that the inclusion of QDs in the PPy film brought additional OH and COOH groups that determined the increase in hydrophilicity. The other two samples, GC/PPy TsOH-QDs urea-PAMAM and GC/PPy TsOH-QDs urea-PAMAM-tTG, have high hydrophilicity.

We were unable to quantify the contact angle between EG and DMSO for the last two modified electrodes due to their high hydrophilicity, so we did not determine the surface energy for these samples. For the GC/PPy TsOH and GC/PPy TsOH-QDs urea, from contact angle measurements, we determined the surface energy via the method described in the supplementary material. These modified electrodes have a surface energy higher than 35 mJ/m^2^. We obtained an increase in the surface energy for the GC/PPy TsOH-QDs urea film. A possible explanation could be the presence of additional functional groups from embedded QDs urea.

**Fig. 4:**
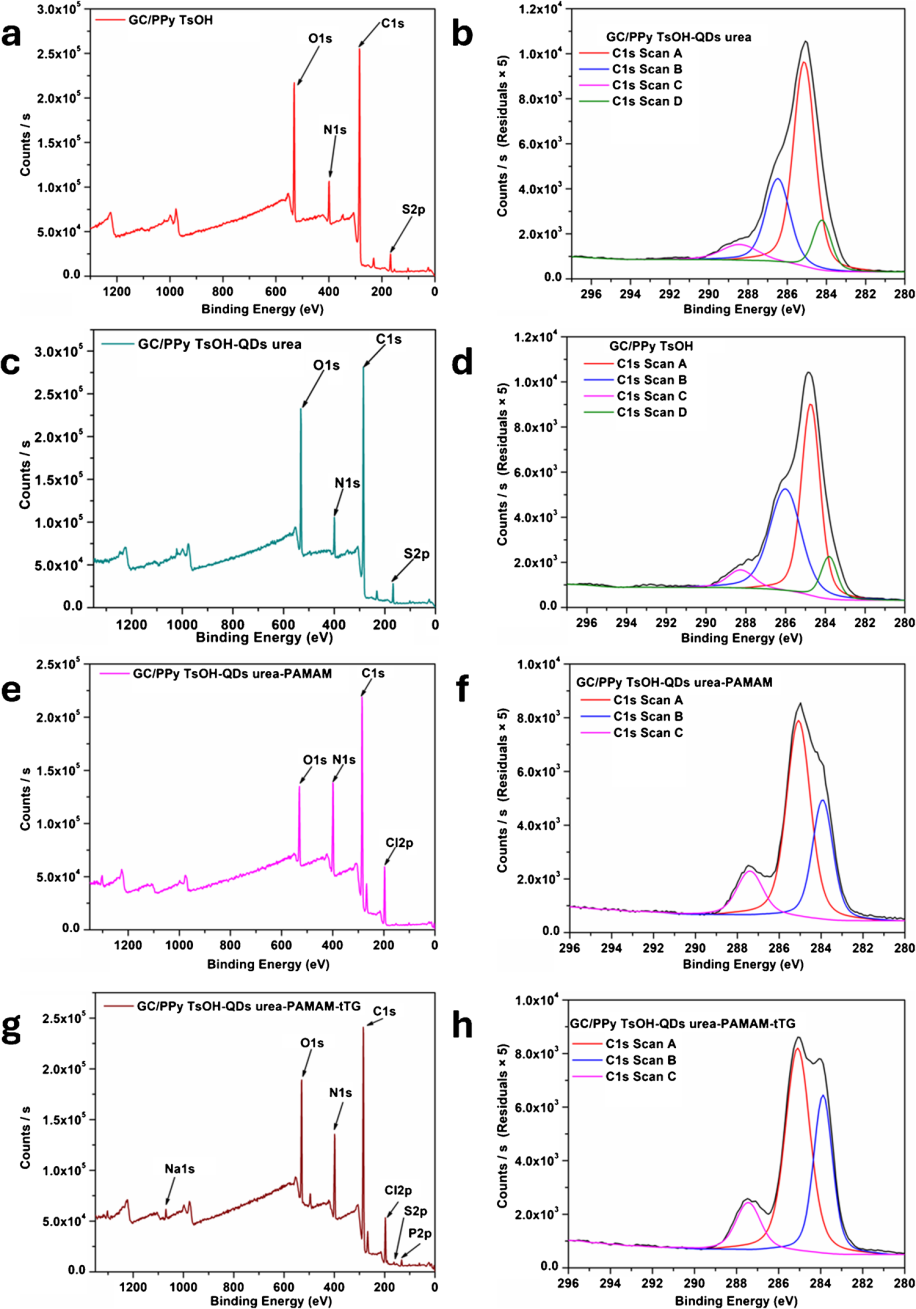
XPS spectra of the modified electrodes. Panels** a**, **c**, **e**, and **g** represent the survey spectrum for each modified electrode, whereas panels **b**, **d**, **f**, and **h** represent the C1s spectrum

A survey of each step of the modification of the glassy carbon electrodes was performed through XPS (Fig. 4). The C1s core-level spectra were also utilized to characterize the electrode surface using XPS following each modification process, revealing the differences in surface chemistry. According to the XPS survey spectra (Fig. 4a, c, e, and g), all the samples exhibit C1s, O1s, N1s, and S2p peaks located at 284.8 eV, 530.3 eV, 398.8 eV, and 163 eV, respectively. In the case of the GC/PPy TsOH-QDs urea-PAMAM and GC/PPy TsOH-QDs urea-PAMAM-tTG samples, a new peak at 197 eV could be seen in the XPS spectra of the samples, and this new peak came from the EDC-NHS solution used to couple the dendrimer onto the electrode surface. The chemical state of C was examined in more detail through deconvolution of the high-resolution C1s XPS spectrum (Fig. 4b, d, f, and h). Four peaks at approximately 283.81 eV, 284.8 eV, 286 eV, and 288.3 eV were assigned to C–S, C = C, C–O, and O–C = O, respectively, and were clearly observed in the GC/PPy TsOH and GC/PPy TsOH-QDs urea samples. For the GC/PPy TsOH-QDs urea-PAMAM and GC/PPy TsOH-QDs urea-PAMAM-tTG samples, the C1s were deconvoluted into three peaks located at 285 eV, 283.79 eV, and 287.36 eV. The peak attributed to O–C = N disappeared because of the covalent bonding of the dendrimer to the electrode surface by the reaction between the NH_2_ groups and COOH groups. Moreover, the percentage of O–C = O groups increased in the GC/PPy TsOH-QDs urea sample compared to that in the GC/PPy TsOH sample (the ratio between O–C = O/C = C was 0.94 in the case of the GC/PPy TsOH sample and 1.64 in the case of the GC/PPy TsOH-QDs urea sample), which indicated that the carbon quantum dots were successfully attached to the electrode surface.

### Electrochemical characterization of modified electrodes

**Fig. 5:**
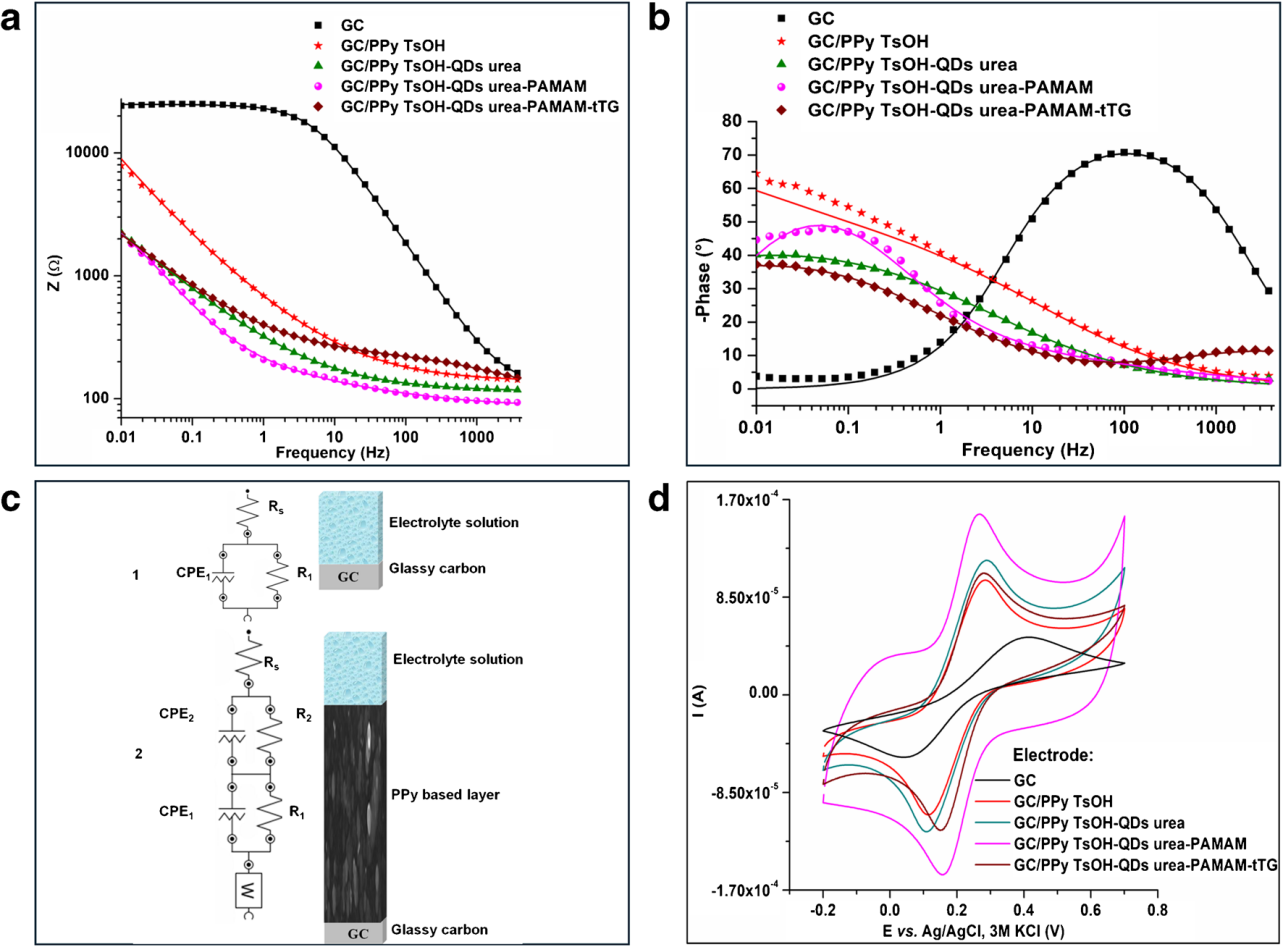
EIS spectra-Bode modulus (**a**) and Bode phase (**b**); CV curves (**c**) corresponding to GC and modified electrodes; in Bode diagrams, the experimental data and fitted curves are shown by dots and solid lines, respectively; in CV, only the second cycle is shown at a 50 mV/s scan rate

Electrochemical impedance spectroscopy (EIS) was used to evaluate the electrical properties and learn more about what is happening at the contact between the liquid and electrode. The results obtained for the GC and modified electrodes are shown in Fig. 5. In Fig. 5a, as the frequency increases, the logarithm of the value of |*z*| decreases. At higher frequencies, the film exhibits resistive behavior. It is evident that the naked GC had a much higher impedance behavior compared to the modified electrodes. The impedance spectra of the GC exhibited a considerable decrease after the deposition of PPy TsOH, which may be attributed to the increased conductivity of PPy TsOH. For the GC/TsOH-QDs urea mixture, the impedance of the porous electrode decreased further. This finding implies that QDs urea enhances the electrochemical activity of PPy TsOH by rapid creation and enhanced transmission of charge carriers. The grafting of PAMAM resulted in an additional reduction in impedance. This could be caused by an increase in the surface area of the nanoporous structure, which decreases the resistance of the modified electrode.

Among the electrodes obtained after each step, GC has the highest phase angle (Fig. 5b). For the modified electrodes, there are phase transitions from the capacitor to the resistor, and the phase angle tends to approach zero at frequencies of approximately 10^4^ Hz. In the high-frequency range, the phase angle shifted upward after tTG immobilization.

Figure 5c shows the equivalent circuits used to fit the impedance data, and the corresponding parameters are given in Table S2 in the supplementary material. Circuit 1 from Fig. 5c is for the GC electrode, and circuit 2 from Fig. 5c is for the modified electrodes. In both circuits, *R*_s_ denotes the electrolyte solution resistance. Instead of using pure capacitance, we opted for a constant-phase element (CPE) because of its nonideal characteristics. The first circuit has CPE_1_ connected in parallel with resistance (*R*_1_) for charge transfer at the GC/electrolyte interface. The second circuit has two parallel pairings of R-CPE. The ionic charge–transfer resistance at the electrolyte-polymer interface is represented by *R*_2_ in parallel with the constant phase element CPE_2_ followed by a Warburg impedance (for semi-infinite diffusion). *R*_1_ in parallel with the constant phase element (CPE_1_) is for the transfer of electrons at the polypyrrole/GC electrode interface.

The Bode plot agrees with the experimental data, as shown by the small relative error in the EIS parameters. Similar resistance values were obtained for the 0.1 M PBS (pH 7.4) with 5 mM K_3_[Fe(CN_6_)] and 5 mM K_4_[Fe(CN)_6_] solutions. Both *R*_1_ and *R*_2_ are lower for the PPy TsOH-QDs urea than for the PPy urea, indicating that the former has greater conductivity (a better charge carrier transfer efficiency). For PPy TsOH-QDs and urea-PAMAM, *R*_1_ and *R*_2_ are the lowest. This suggested that grafting the PAMAM dendrimer increased the porosity of the surface (as also observed in the SEM images), which increased the area of the surface that the electrolyte could reach. Similarly, *R*_1_ was obtained for all electrodes containing PPy TsOH-QDs urea. PPy TsOH-QDs urea-PAMAM-tTG showed higher *R*_1_ and *R*_2_ values than did PPy TsOH-QDs urea-PAMAM. An increase in resistance after enzyme immobilization with a cross-linker was also observed in another study because bioactive compounds (enzyme and cross-linker) may reduce the accessibility of the electrochemical probe and slow interfacial electron transfer [31].

Pseudocapacitive behavior is shown for every sample for the polypyrrole/GC electrode interface since the values of *N*_1_ values are greater than 0.5.

Figure 5d shows a comparison of the [Fe(CN)_6_]^3−/4−^ redox probe voltammograms obtained after each step of the GC electrode modification. All the electrode modifications gave rise to higher peak currents for the ferricyanide oxidation/reduction reaction than for the bare GC. The PPy TsOH-QDs urea-PAMAM-modified electrode exhibited the highest peak current for the oxido-reduction of ferrocyanide ions. This observation indicates an increased surface area of the electrode. The PPy TsOH-QDs urea-PAMAM-tTG-modified electrode exhibited lower peak currents than did the PPy TsOH-QDs urea-PAMAM electrode. The same trend was also observed following the immobilization of another enzyme onto the matrix surface by covalent attachment of the cross-linker [31]. This can be due to the nonconducting character of both the enzyme and the cross-linker, which slows the electron transfer process [31]. This indicates the effective immobilization of the enzyme on the electrode surface.

As shown in Fig. 5a, compared with PPy TsOH, PPy TsOH-QDs urea and PPy TsOH-QDs urea-PAMAM had a proportional drop in resistance, and they also had an increase in current (Fig. 5d). The EIS and CV findings are in good agreement.

The presence of p-type conductance is suggested by the negative slope of the Mott–Schottky plot for all the modified electrodes (Fig. S5 from the supplementary material). The plots exhibit a noticeable curvature at − 0.6 V. Similar curvatures have been observed in Mott–Schottky plots in relation to poly(pyrrole-*N*-propionic acid) and other electrodes utilized for electrochemical charge storage [32, 33]. One plausible explanation is that when potentials lower than the flatband potential are applied, the Fermi level of the PPy semiconductor is compelled to increase. Consequently, the bands incline downwards to align with the electrolyte’s Fermi level, facilitating electron transfer from the electrolyte to the semiconductor. This process results in the filling of electron vacancies in the valence band, generating a negative charge within the semiconductor. This negatively charged region lacks vacancies, which are the principal charge carriers in a p-type semiconductor, thus forming a space-charge or depletion area. The formation of a depletion layer is closely associated with the elevated value observed in the Mott–Schottky plot utilizing the Excel software [33].

The charge carrier density values obtained are shown in Table S3 in the supplementary material. The GC/PPy TsOH-QDs urea electrode showed the highest charge carrier density.

To analyze the redox process occurring on the modified electrodes, the influence of the scan rate on the [Fe(CN)_6_]^3−/4−^ redox probe was investigated using the modified electrodes obtained in each step. In all the cases, an increase in the peak current with increasing scan rate is observed due to the decreasing size of the diffusion layer. Simultaneously, as the scan rate increased, the quasireversibility of the process occurring at the electrode surface was further validated by the increase in peak separation potential (Δ*E*) caused by a positive shift in the anodic peak potential and a negative shift in the cathodic peak potential. The dependency of the peak current on the square root of the scan rate showed a linear trend for all the types of modified electrodes, which suggested a quasi-reversible diffusion process for each modification step, as shown in Fig. S6.

The diffusion coefficient (*D*) was calculated according to the methods provided in the supplemental material and was found to be 3.945·10^−6^ cm^2^/s. With this value, the electrochemically active surface area was calculated using the Randles–Scevcik equation for quasi-reversible systems (see supplementary material) for each modification step. The resulting values were as follows: 0.139 cm^2^ for GC/PPy TsOH, 0.162 cm^2^ for GC/PPy TsOH-QDs urea (a 17% augmentation), 0.308 cm^2^ for GC/PPy TsOH-QDs urea-PAMAM (a remarkable 122% increase), and 0.147 cm^2^ for GC/PPy TsOH-QDs urea-PAMAM-tTG.

### Reproducibility and stability

**Fig. 6:**
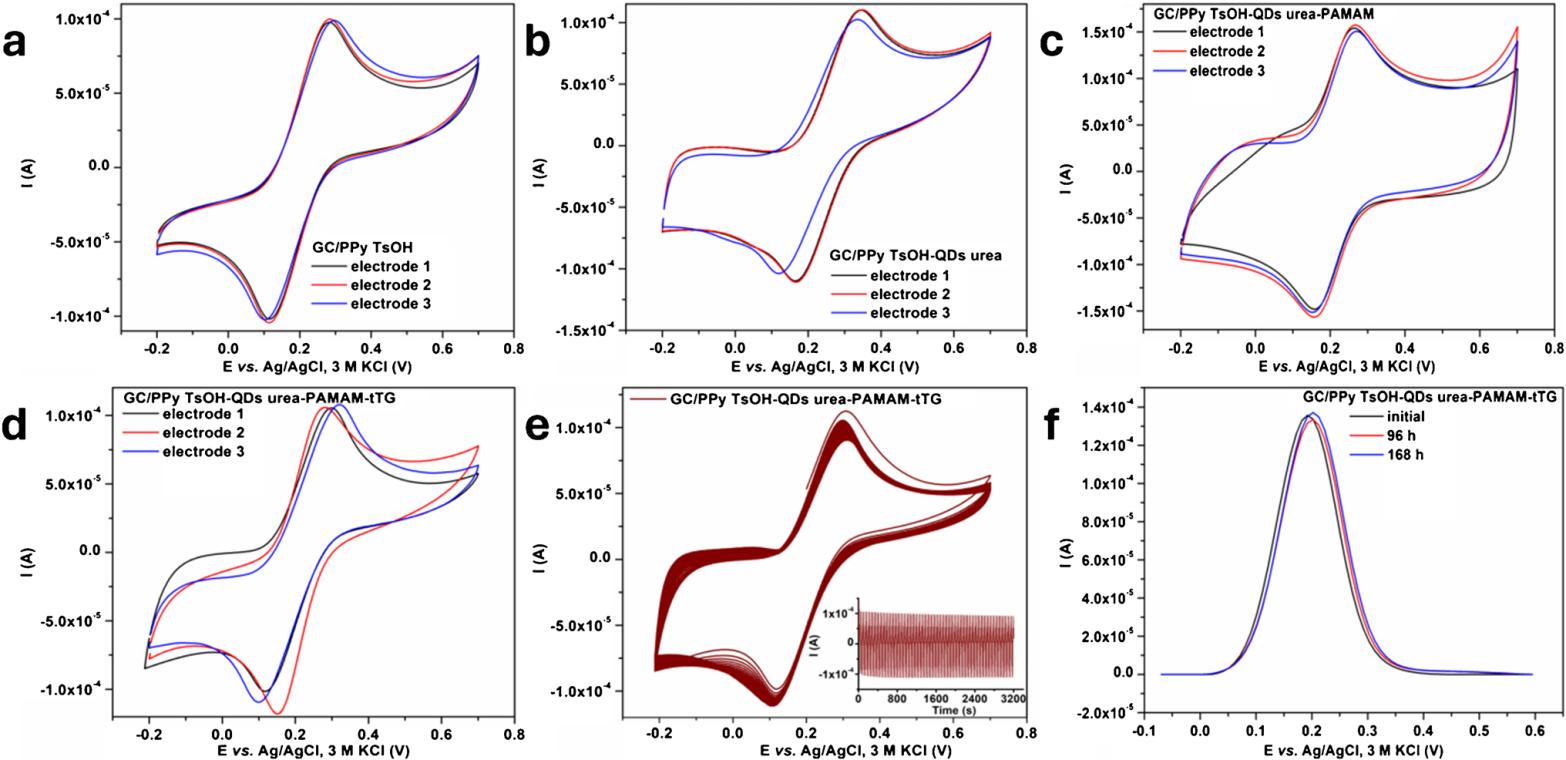
CV curves and second cycle for 3 freshly prepared electrodes: GC/PPy TsOH (**a**), GC/GC/PPy TsOH-QDs urea (**b**), GC/PPy TsOH-QDs urea-PAMAM (**c**), GC/PPy TsOH-QDs urea-PAMAM-tTG (**d**), 100 cycles of GC/PPy TsOH-QDs urea-PAMAM-tTG (**e**), and DPV for GC/PPy TsOH-QDs urea-PAMAM-tTG at different times (**f**). The curves were recorded for 5 mM Fe^2+^/Fe^3+^ in PBS

In addition to their analytical performance, practical considerations should also be considered for modified electrodes, namely, the repeatability of their preparation and stability over time. Thus, the reproducibility of three freshly prepared electrodes in each step was checked by performing CV in PBS containing [Fe(CN)_6_]^3−/4−^ as a redox probe. Figure 6 displays the obtained results for the second cycle (50 mV/s scan rate). In all the cases, the modified electrode obtained after each step showed similar responses toward the redox probe, which indicated very good reproducibility of the elaboration process. The relative standard deviation (RDS) for the oxidation peak of each of the four modified electrodes was as follows: 1.08%, 3.97%, 2.19%, and 1.12%. For the reduction peak, RSDs of 1.27%, 3.48%, 2.80%, and 7.51% were obtained.

The stability of the GC/PPy TsOH-QDs urea-PAMAM-tTG electrode was tested by performing 100 consecutive CV cycles (50 mV/s scan rate). Figure 6e shows that the electrode gave a stable signal. A decrease of 16.18% in the oxidation peak current intensity was obtained after 100 cycles, while for the reduction peak current, the decrease was 7.3%.

Stability over time is another crucial factor that influences the use of modified electrodes, particularly those based on enzymes. To verify this, three identically prepared GC/PPy TsOH-QDs urea-PAMAM-tTG electrodes were used to evaluate the [Fe(CN)_6_]^3−/4−^ redox probe signal. One electrode was tested on its preparation day, and the other two were kept at 4 °C and tested after 96 and 168 h. As shown in Fig. 6f, the signal remains at 95% of its initial value even after 168 h.

The results obtained conclusively show that the GC/PPy TsOH-QDs urea-PAMAM-tTG electrodes have sufficient stability over time, enabling them to be applied in practical applications.

### Antibody level determination with modified electrode using DPV

**Fig. 7:**
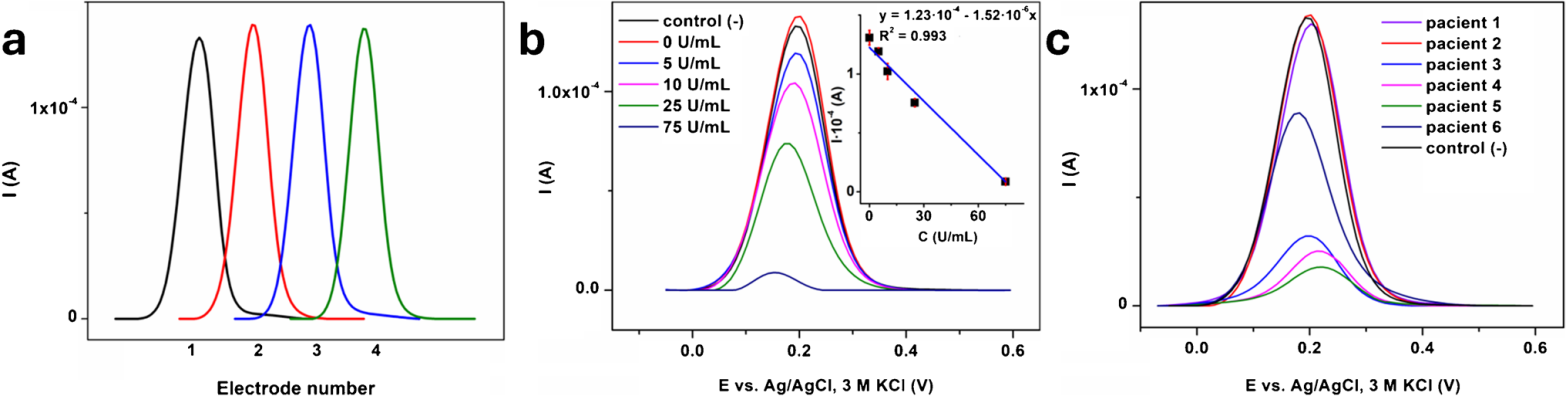
DPV curves corresponding to the GC/PPy TsOH-QDs urea-PAMAM-tTG electrode after 1 h of incubation in a 10^−4^ mg/mL commercial antibody (4 freshly prepared electrodes) (**a**); GC/PPy TsOH-QDs urea-PAMAM-tTG electrode after 1 h of incubation in commercial antibody solutions with different concentrations with the corresponding calibration curve (**b**). The curves were recorded for 5 mM [Fe(CN)_6_]^3−/4−^ in PBS

To ensure reproducibility, four GC/PPy TsOH-QDs urea-PAMAM-tTG electrodes were prepared and incubated for 1 h at room temperature in a PBS solution containing 10^−4^ mg/mL commercial antibody and then scanned in PBS containing an [Fe(CN)_6_]^3−/4−^ redox probe. An RSD of 2.07% was obtained, which demonstrated the effectiveness of the modified electrode preparation and the repeatability of the results (Fig. 7). The response of the modified electrode toward the most prevalent proteins found in the bloodstream (albumin, γ-globulin, and creatinine) that may potentially impede the detection of anti-tTG antibodies has also been scrutinized (refer to Fig. S7 in the supplementary material). These proteins do not manifest a significant impact on the initial electrode signal (relative standard deviations of 2.03%, 2.63%, and 1.57% were acquired for albumin, γ-globulin, and creatinine, respectively), aligning with the preceding research [8], and the references therein.

The response of the GC/PPy TsOH-QDs urea-PAMAM-tTG electrode was further examined in the presence of commercial anti-tTG antibodies (Fig. 7b) by DPV measurements between − 0.1 and 0.6 V (vs. Ag/AgCl 3 M KCl reference electrode). All measurements are performed in triplicate, and the average value for each antibody concentration was used for the calibration curve. The signal exhibits direct proportionality to the concentration so that an increase in concentration results in a reduction in the signal. A linear relationship was observed between the peak current height and concentration when plotting the data for the modified electrode incubated for 1 h in target anti-tTG antibodies and then scanned in [Fe(CN)_6_]^3−/4−^. The linear response range was found to be 0–75 U/mL, with an *R*^2^ value of 0.993, as shown in the inset of Fig. 7b, the standard deviation being 0.0624 for 0 U/mL, 0.0308 for 5 U/mL, 0.06721 for 10 U/mL, 0.03386 for 25 U/mL, and 0.00528 for 75 U/mL. Utilizing the Excel software, the LOD was computed using the formula 3.3·*σ*/*S*, where *S* represents the slope of the calibration curve and *σ* denotes the residual standard deviation of the response. This method was chosen as a superior means of LOD determination in comparison to relying solely on the mean blank value [34, 35]. The estimated detection limit was 0.79 U/mL. The detection limit obtained in another study by amperometric detection with gold electrodes covalently attached to tTG was 390 ng/mL [7]. Using AuNPs electrodeposited on overoxidized PPy and voltammetry, Natasha West and colleagues [36] achieved a detection limit of 5.2 ng/mL.

The immunosensor developed in this study demonstrates comparable analytical performances, and in certain instances, even greater sensitivity than other electrodes for the detection of anti-tTG antibodies (refer to Table 1). It is evident that a direct electrochemical label like QDs can rival enzymatic labels. However, it is worth noting that not all studies report their findings using consistent units of measurement for anti-tTG IgG antibodies, making it challenging to compare the analytical characteristics of various biosensors, particularly when an enzymatic label is involved.

**Table 1:** The limit of detection for the voltammetric detection of anti-tTG antibody that was attained with different electrodes

Nature of electrode	Linear domain	Limit of detection	Reference
SPCE^a^-tTG	0–40 U/mL	2.2 U/mL	[8]
SPCE–MWCNT^b^–NPAu^c^	0–40 U/mL	Not specified	[6]
Dual SPCE–MWCNT–NPAu/gliadin (WE1)/tTG solution (WE2)	0–100 U/mL	2.45 U/mL	[9]
Au self-assembled monolayer-tTG	0–10 µg/mL	390 µg/mL	[8]
Au-overoxidized polypyrrole nanocomposite	10^−6^–10^−4^ mg/mL	5.2 ng/mL	[34]
Gold nanoelectrode ensembles	0.005–1 µg/mL	1.8 ng/mL	[37]
Gold screen-printed electrode (SPE)-attached DNA oligomer	0.01–10 U/mL	10^−2^ U/mL	[38]
Gold nanoelectrode ensembles	0.25–8.54 U/mL	0.72 U/mL	[3]
GC/PPy TsOH-QDs urea-PAMAM-tTG	0–75 U/mL	0.79 U/mL	This work

^a^*SPCE*, carbon screen-printed electrode

^b^*MWCNT*, multi-walled carbon nanotubes

^c^Gold nanoparticles

The methodologies we propose offer the advantage of obviating the need for a secondary antibody or additional enzymes, unlike the other studies mentioned in Table 1 that require an additional step to conduct the bioassay. Similar to other electrochemical detection techniques, our approach requires only a minute amount of sample (specifically, just 6 µL in our case) to initiate the detection process, which can be completed in a matter of minutes. Nonetheless, our method utilizes traditional solid electrodes known for their consistent performance, albeit requiring meticulous attention to ensure proper cleaning. In contrast, some researchers in Table 1 utilize screen-printed electrodes, which offer the advantage of being portable and easily deployable devices.

The electrochemical sensors outlined in Table 1 exhibit remarkable sensitivity in detecting minute concentrations of antibodies. While they are adept at discerning these crucial antibody levels within the range of 0–10 U/mL, only a select few electrochemical detection techniques, including our own research and [9], have demonstrated the ability to detect heightened antibody levels. None of these methodologies presently possesses the capacity to identify antibody concentrations up to 200 U/mL, a capability currently exclusive to the ELISA test. Consequently, it is evident that further advancements are imperative in this domain.

## Conclusions

This work assessed the efficacy of using a PPy film that has been enhanced with PAMAM G4 and tTG protein as a sensing platform for anti-TG antibodies specific in CD. By incorporating the conducting PPy with the prepared carbon nanostructures onto the sensing surface of a GC electrode, we managed to obtain a low detection limit for anti-tTG antibodies, eliminating the need for an amplification step. The modified electrode could allow the rapid detection and monitoring of CD, even in the presence of low antibody levels, using a small amount of sample without the need for a secondary antibody. It is straightforward to prepare and use inexpensive raw ingredients, including citric acid, urea, and pyrrole.

Additionally, this study demonstrated that the electro-polymerization process of pyrrole monomer in the presence of TsOH created a uniform and porous layer with nanostructures that were arranged hierarchically and facing upward being different from the usual cauliflower-like shape. The conjugation of carbon nanostructures obtained from citric acid and urea with PPy enhances the active surface area and enhances the electrical characteristics of the layer.

The newly obtained modified nanohybrid GC/PPy TsOH-QDs urea-PAMAM-tTG electrode was synthesized under consistent conditions with demonstrated stability for a period of 7 days. It is capable of distinguishing between the key concentrations (0–10 U/mL) of anti-tTG antibodies, with the detection limit exhibiting LOD levels below the diagnostic threshold. Nevertheless, enhancements are necessary to facilitate the identification of exceedingly high antibody levels (200 U/mL) found in certain patients. Additionally, it may be advantageous to modify the technique for use with screen-printed electrodes to develop a portable and easily deployable device.

## Supplementary information

Below is the link to the electronic supplementary material.

Supplementary file1 (2.41 MB)
