# Supplementary material for: Electrochemical detection of anti-tissue transglutaminase antibody using quantum dots-doped polypyrrole-modified electrode
Source: Mikrochim Acta. 2024 Aug 17;191(9):543. doi: 10.1007/s00604-024-06620-w (PMC11330391; doi:10.1007/s00604-024-06620-w)
Supplement: Supplementary file 1 — Supplementary file1 (2.41 MB) [file 604_2024_6620_MOESM1_ESM.docx]

**SUPPORTING INFORMATION**

Electrochemical detection of anti-tissue transglutaminase antibody using quantum dots-doped polypyrrole-modified electrode

Cristina Dumitriu^a^, Andreea Madalina Pandele^a^, Mihaela Vasilica Mîndroiu, Oana-Andreea Lazar^c^, Alina Popp^b^, Marius Enachescu^c,d^, George Octavian Buica^a,⁎^

^a^National University of Science and Technology Politehnica Bucharest, 313 Splaiul Independentei, Sector 6, Bucharest 060042, Romania

^b^National Institute for Mother and Child Health “Alessandrescu-Rusescu”, 120 Lacul Tei Boulevard, Sector 2, Bucharest 020395, Romania

^c^Center for Surface Science and Nanotechnology, National University of Science and Technology Politehnica Bucharest, 313 Splaiul Independentei, Sector 6, Bucharest 060042, Romania

^d^Academy of Romanian Scientists, Splaiul Independentei 54, Bucharest 050094, Romania

**Equipment and methods**

A Perkin-Elmer L950 UV‒Vis/NIR spectrophotometer was used for the UV‒VIS spectral investigations. An integrating sphere accessory from this equipment was used for solid sample examination. A Perkin-Elmer Spectrum 100 Fourier transform infrared spectrometer was used to obtain the FT-IR spectra. Four sequential scans with a resolution of 4 cm^−1^ were carried out between 4000 and 600 cm^−1^. A quartz cuvette (with a 1 cm path length) was used to measure the fluorescence spectra using a JASCO FP-6500 spectrofluorometer at room temperature. The emission and excitation silts were fixed at 5 and 10 nm, respectively. At least three replications of each determination were made. Using a KSV CAM100 optical contact angle and surface tension meter, contact angle data were recorded. The sessile drop technique was used, with an image analysis system and a photo camera used to store the drop image. The drop shape was used to compute the contact angle (θ). A 3-5 μL liquid droplet formed and settled over the surface. At least three contact angle (θ) values were recorded for each measurement, and the mean value and standard deviation were computed using the Excel program.

With the use of a Hitachi SEM SU8230 scanning electron microscope, SEM images and EDX spectra were acquired. Furthermore, electron images were recorded by an electron beam with different acceleration voltages, 10 kV and 5 kV. A Hitachi HD 2700 high-resolution scanning transmission electron microscope (HR-STEM) was used to study the morphological and topographical properties of the produced nanoparticles (NPs). Standard Cu TEM grids with a thin layer of carbon sheets were used to deposit the liquid sample for examination. The signals collected for the measurements were as follows: secondary electron microscopy (SEM) images, which provided topographic information; incoherent elastic (Rutherford) scattered electrons, which provided compositional contrast (Z-contrast or HAADF images); and transmitted electrons, which were used for internal structure characterization (TEM images).

A Thermo Scientific K-Alpha spectrometer equipped with monochromatic Al Kα X-rays (1486.6 eV) at a 90° take-off angle was used to perform X-ray photoelectron spectroscopy (XPS). Resolutions of 200 eV and 20 eV were used for the survey and high-resolution spectra, respectively. Shirley background removal was carried out before using a mixed Gaussian Lorentzian function for the deconvolution of core-level spectra.

To prepare or characterize the modified electrodes, electrochemical methods were performed with Nova software, version 1.11, and an Autolab Potentiostat/Galvanostat PGSTAT302N from Metrohm, BV. For electrochemistry, a three-electrode cell with an Ag/AgCl 3 M KCl reference electrode and a platinum rod counter electrode was used. These were all bought from Metrohm, BV. The measurements were performed at room temperature. In all the cases, glassy carbon (GC) working electrodes (3 mm in diameter) were used. The electrolyte used for electrode modifications will be mentioned for each patient. For electrochemical characterization and detection, 0.1 M PBS (pH 7.4) with 5 mM K_3_[Fe(CN_6_)] and 5 mM K_4_[Fe(CN)_6_] was used. PBS was prepared from 0.2 M stock solutions of NaH_2_PO_4_ and Na_2_HPO_4_. At the open-circuit potential, the electrochemical impedance spectroscopy (EIS) parameters used ranged from 3700 to 0.27 Hz, and the amplitude was 0.01 V. Experimental differential pulse voltammetry (DPV) measurements were taken at a rate of 20 mV/s between -0.1 V and 0.6 V or 0.8 V (against Ag/AgCl, 3 M KCl), with an amplitude of 50 mV, 10 mV step potential and pulse duration of 0.5 s. For cyclic voltammetry (CV), the voltage was varied within the range of -0.2 to 0.7 V or -0.1 to 0.6 V (against Ag/AgCl, 3 M KCl) with a 2 mV step and a scan rate of 50 mV/s. Additionally, CV recordings at different scan rates between 20 and 200 mV/s were performed. A frequency of 1000 Hz was used for the Mott–Schottky analysis. The experiment included measuring the impedance of the samples at potentials ranging from -0.6 to 0.85 V (against Ag/AgCl and 3 M KCl) in increments of 50 mV with a 10 mV amplitude.

**Carbon Quantum dots (QDs) synthesis**

**QDs citric, QDs folic**, **QDs cys and QDs urea** were prepared and characterized.

**QDs citric solution**. According to literature, the citric acid can be heated to a point where it decomposes; the resulting hydronium ion then serves as a catalyst for the next phases of the decomposition process. By using aldol condensation and cycloaddition, aromatic clusters are formed, leading to aromatization [1]. Under basic conditions, CA self-assembles into a sheet structure [2], so GQDs are then generated by adding sodium hydroxide [1]. It has been shown that the development of GQDs is most facilitated by a pH of 9 [1]. We prepared them according to the procedure reported in literature using the pyrolysis approach [3]: 5 g of citric acid monohydrate powder was deposited in a ceramic crucible and subjected to thermal treatment at 200°C for 30 minutes, in an air electric furnace. With 1.5 M NaOH, the melted dense solution was brought down to pH 9.0, and an aqueous solution of GQDs was made. The solution was filtrated through a microporous membrane (0.22 μm) to remove the large particles and kept in the fridge at 4 °C.

**QDs folic solution**. According to the study [4], N-doped dots could be made by carbonizing and polymerizing folic acid. Folic acid is high in nitrogen and has functional groups like -OH, -NH_2_, and -COOH. Very few additional additives or passivation agents would be needed for this process [4]. We prepare these nanostructures according to a method described in literature [4] with some modifications: folic acid (0.0050 g) was dissolved in 7.5 mL of ultrapure water. The solution was sonicated to form a well-distributed solution and then heated in a Teflon-lined stainless-steel reactor (50 mL) for 6 hours at 220 °C for carbonization. After this reaction, a clear, light yellow-brown solution was obtained. Filtering with 0.22 μm filters further purified the solution, which was kept in the fridge.

**QDs cys solution**. Because of its abundance of S and N, Cys is a promising bio-source and building block for S- and N-doped QDs [5]. A one-step hydrothermal process was used to easily manufacture sulfur and nitrogen codoped graphene quantum dots, as reported in the literature [6]. We used for synthesis a facile hydrothermal route described in the literature [6] with some modifications : a mixture of 12 g of citric acid (for carbon) and 1.2 g of L-cysteine (for nitrogen and sulfur) was ultrasonicated with 30 milliliters of ultrapure water. After that, 50 mL of the mixed solution was moved to a Teflon-lined stainless-steel reactor (50 mL) and heated at 200 °C for 8 h. The reactor was allowed to come down to room temperature after the reaction. Using 0.22 μm filters allowed for further purification of the brown-yellow solution and no other purification methods were applied. Solution was stored at 4°C in the fridge.

**QDs urea** solution (nitrogen-doped carbon quantum dots) were prepared according to a reported route [7] with small modifications: 2 g of citric acid monohydrate powder and 0.1 g of urea were dissolved in 30 mL of ultrapure water (Millipore) using ultrasound, then transferred to a Teflon-lined stainless-steel reactor (50 mL). Six hours of heating at 200 °C was applied in an electric oven for carbonization. To eliminate the larger particles, the solution underwent filtration through a microporous membrane (0.22 μm) once the reactor had naturally cooled to room temperature, and further utilized as such. Solution was stored at 4°C in the fridge.

**GC/PPy TsOH-QDs citric, GC/PPy TsOH-QDs folic and GC/PPy TsOH-QDs cys** were prepared from 0.2 M pyrrole monomer aqueous solution with 0.1 M TsOH electrolyte adding in each case 7.5 mL QDs solution/25 mL electrolyte solution.

**QDs spectroscopical characterization**

Citric acid, a small and aromatic molecule, was used because it is cheap, and carbon quantum dots can be prepared via the facile carbonization process that involves the conversion into carbon through pyrolysis distillation [1, 8]. The use of heteroatoms as dopants in carbon quantum dots has shown its efficacy in modifying the band gap, adjusting electronic density, and manipulating chemical activity of QDs [9]. Due to the comparable atomic size of nitrogen and carbon, as well as its electron-donating nature [7], the addition of nitrogen atoms to the structure of carbon quantum dots not only helps to increase quantum yields, but it also helps to improve the electrocatalytic activity [9]. We used urea or folic acid for nitrogen doped QDs. Additionally, the S atom is extensively utilized for doping QDs. Doping with S may successfully alter the electronic structure of QDs by injecting energy levels associated to S between π and π* of C, resulting in efficient and numerous emission peaks [9]. Aiming to take advantage of simultaneously N and S doping, we used cysteine.

FT-IR, UV-Vis and Florescence spectra of each QDs sample are presented in Fig. 1S.

The UV-VIS spectral investigation was carried out utilizing a Perkin-Elmer L950 UV-Vis/NIR spectrophotometer and quartz cuvettes. Quantum dots solutions were appropriately diluted. The Perkin-Elmer Spectrum 100 Fourier transform infrared spectrometer was utilized to capture FT-IR spectra, by carefully depositing a few microliters of solution onto the analyzer using a micropipette. Four consecutive scans were conducted within the range of 4000 to 600 cm^-1^, each with a precision of 4 cm^-1^. Fluorescence spectra were analyzed at ambient temperature employing a JASCO FP-6500 spectrofluorometer and a quartz cuvette with a path length of 1 cm. The excitation and emission slits were set at 10 and 5 nm, respectively. Three determinations were replicated for accuracy.

In QDs citric FT-IR spectra (Fig. 1S a), presents a strong and widened peaks appear at 3000-3500 cm^−1^ (attributed to O-H stretching). The C=O (from COOH) stretching caused the 1770 cm^-1^ peak [8, 10]. The absorption peak at 1683 cm^-1^ indicates C=O band stretching vibration [6]. An aromatic C=C stretching peak at 1576 cm^−1^ indicates dehydration of Citric acid to GQDs [11]. A peak at 1397 cm^-1^ can indicate the stretching vibration of the -COO bonds [12]. The stretching vibration of C-O-C at 1250 cm^−1^ indicates that GQDs may include partially carbonized citric acid [11]. The peak at 1075 cm^−1^ can be attributed to stretching vibrations of C–OH bonds [13].

In the FT-IR spectra of QDs folic (Fig. 1S d), the stretching vibration of C=O is responsible for the peak at 1634 cm^-1^ [14]. At 1450 cm^−1^, the saturated C-H bending vibration was identified [7]. The peak at 1316 cm^-1^ is the result of C-H, O-H, and C-N groups undergoing deformation [15]. The band at 1245 cm^-1^ is the result of vibrations caused by C-N stretching [15].

In the FT-IR spectra of QDs urea (Fig. 1S g), C=O stretching (from -COOH) is responsible for the peak at 1790 cm^-1^ [8]. The stretching vibration of C=O is responsible for the peak at 1635 cm^-1^ [14]. It is shown that citric acid has fully dehydrated to GQDs by the presence of an aromatic C=C stretching peak at 1570 cm^−1^ [11]. The saturated C-H bending vibration was linked to the peak at 1457 cm^−1^ [7]. The observed peak at a wavenumber of 1100 cm^−1^ may be ascribed to the occurrence of C-N bond formation [10]. The 1025 cm^−1^ peak is the result of C-O stretching [11].

The FT-IR spectra of QDs cys (Fig. 1S j) show a faint distinctive signal at 2600 cm^-1^, which is induced by stretching vibrations of the -SH group [16]. The peak at 1706 cm^-1^ may be attributed to C=O stretching, which originates from -COOH [8]. At 1634 cm^-1^, the stretching vibration of C=O is responsible for the peak [14]. The peak at 1525 cm^−1^ can be attributed to aromatic C=C stretching [17]. The peak at 1400 cm^-1^ may correspond to the C–N stretching vibration [14]. Stretching vibration of C-S was supposed to be responsible for the peak at 1218 cm^−1^ [5].

In the UV-VIS spectra, a peak appears at around 350 nm (strong for QDs citric and QDs cys, Fig. 1S b, k; weak for QDs urea, Fig. 1S h). For QDs folic, it is at around 292 nm (Fig. 1S e). This peak can be attributed to the surface states trapping the excited state energy – the band-gap transition n-π* of C=O and/or C=N [10, 15]. For QDs folic and QDs urea samples, the presence of a peak at between 200 and 250 nm is observed (Fig. 1S h, e). The observed peak might perhaps be attributed to the π -π* transition of C=C in the aromatic structure [10, 15]. This observation suggests the existence of functional groups such as carboxyl groups and other electron pairs that are separated [14].

For QDs citric an intense fluorescence emission peak can be seen at ~ 473 nm wavelength at the excitation wavelength of 360 nm. This is similar with what Jaya Prakash Naik and coworkers reported [1]. QDs folic has fluorescence peak at ~ 400 nm at the excitation wavelength of 360 (Fig. 1S f), consistent with the findings of other scientific investigations [12, 18].The QDs urea (Fig. 1S i) has a emission peak at 450 nm at the excitation wavelength of 360 nm, similar with observations made in other study [7]. In the fluorescence spectra of QDs cys (Fig. 1S l) an emission peak can be seen at ~ 450 nm wavelength at the excitation wavelength of 400 nm, as also observed in literature [6, 19].


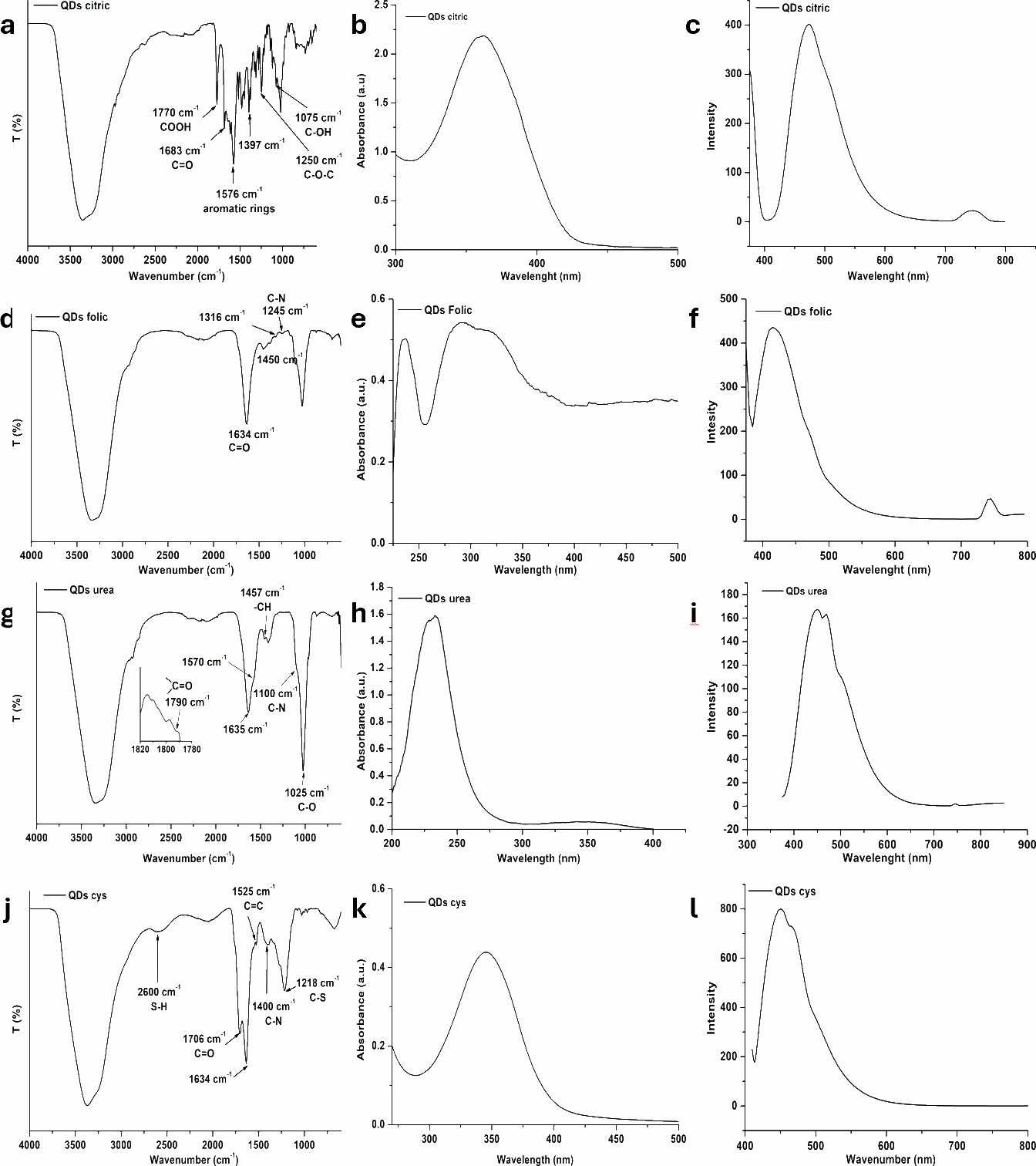


Fig. S1 QDs spectra: a, b and c – FT-IR, UV and Fluorescence spectra for QDs citric; d, e and f – FT-IR, UV and Fluorescence spectra for QDs folic; g, h and i – FT-IR, UV and Fluorescence spectra for QDs urea; j, k and l – FT-IR, UV and Fluorescence spectra for QDs cys.

**GC electrode modification with PPy and PPy/QDs films**

Fig. S2 a clearly shows that all synthetized QDs can be used for doping PPy for an enhanced DPV signal. The GC/PPy TsOH-QDs urea electrode has the highest peak height at around 0.2 V when performing a DPV in a solution of 0.1 M PBS pH 7.4 containing 5 mM K_3_[Fe(CN_6_)] and 5 mM K_4_[Fe(CN)_6_]. To gain more information, UV-VIS spectra were also recorded, and using Excel calculations, we were able to make the comparative Tauc plot for GC/PPy TsOH and GC/PPy TS OH-QDs urea modified electrodes. Figure 2S b shows the Tauc plots that were used to find the band gap energy. Fig. 2S c shows the Urbach energy.


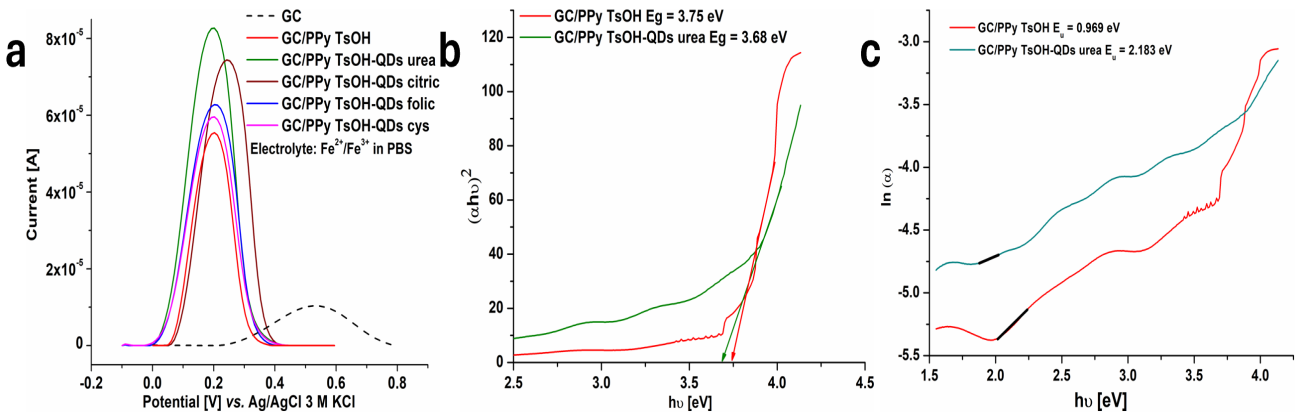


Fig. S2 a) DPV curves for different PPy-QDs films compared to GC and GC/PPy TsOH; b) Tauc Plot for GC/PPy TsOH and GC/PPy TsOH-QDs urea; c) Urbach energy diagram for GC/PPy TsOH and GC/PPy TsOH-QDs urea.

**QDs urea and modified electrodes surface characterizations**

Elemental mapping of modified electrodes can be observed in Fig. S3.


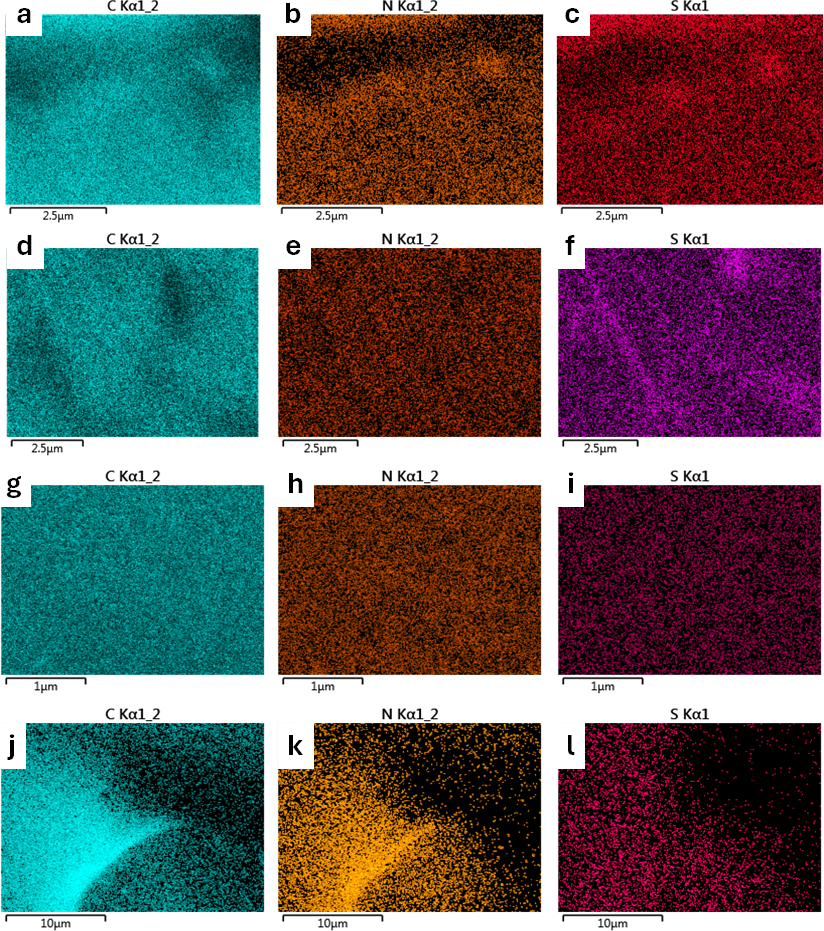


Fig. S3 Distribution of C, N, S determined in elemental mapping analysis for: GC/PPy TsOH (a), (b), and (c); GC/PPy TsOH-QDs urea (d), (e), and (f); GC/PPy TsOH-QDs urea-PAMAM (g), (h), and (i); GC/PPy TsOH-QDs urea-PAMAM-tTG (j), (k), and (l).

It was shown that the use of a pre-existing hydrophilic nanocoating layer prior to the sensitive structure would enhance the diffusion of water molecules, as well as the movement of ions inside the multilayer structure [20]. In these considerations, contact angle measurements were taken for each electrode modification step, and surface energy was determined for the first two samples and they are presented in Table S1. It was observed that the formation of polarized ions, such as carboxyl (-COOH), carboxylate (COO−), and hydroxyl (OH), on surface molecules is the primary cause of the increase in surface energy [21]. Hydrophilic functional primary and tertiary groups (–NH_2_) present in highly branching and well-defined three-dimensional PAMAM macromolecules [22, 23] and tTG which is made up of 687 amino acids [24].

Using the Owens and Wendt method described in other studies [25] we determined surface energy. Values obtained in this study are similar with surface energy values obtained for PPy spherical nanoparticles [26]. Research has shown that PPy with aromatic group dopants had lower surface energy values than PPy with the longest alkylic chain dopant [27]. Our findings are consistent with past studies showing that samples coated with reduced graphene oxide - PPy are hydrophilic and have a greater surface energy than those coated with PPy alone [28].

Table S1. Contact angle and surface energy.

| **Sample** | **Contact angle**  **(°)** | | | **Surface energy**  **[mJ/m^2^]** |
| --- | --- | --- | --- | --- |
|  | **Water** | **EG** | **DMSO** |  |
| GC/PPy TsOH | 82±0.7 | 49±0.2 | 26±0.1 | 40 |
| GC/PPy TsOH-QDs urea | 56±2.3 | 43±0.3 | 45±1.9 | 44 |
| GC/PPy TsOH-QDs urea-PAMAM | 13±0.89 | - | - | - |
| GC/PPy TsOH-aQDs urea-PAMAM-tTG | 11±1.83 | - | - | - |

**Modified electrodes electrochemical characterizations**

**
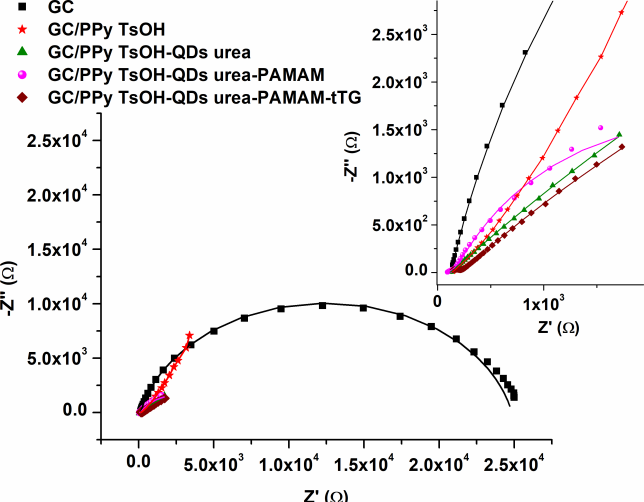
**

Fig. S4 Nyquist diagram corresponding to GC and modified electrodes

Table S2. The numerical values for the equivalent circuit components related to GC and modified electrodes in each step obtained from fitting the EIS experimental data.

| Parameter | | Sample | | | | |  |
| --- | --- | --- | --- | --- | --- | --- | --- |
|  |  | GC | GC/PPy TsOH | | GC/PPy TsOH-QDs urea | GC/PPy TsOH-QDs urea-PAMAM | GC/PPy TsOH-QDs urea-PAMAM-tTG |
| R_s_ (Ω·cm^2^) | | 124.1 | 137.85 | | 114.15 | 84.222 | 94.164 |
| R_1_ (Ω·cm^2^) | | 2.471×10^4^ | 0.921×10^4^ | | 0.447×10^4^ | 0.363×10^4^ | 0.287×10^4^ |
| CPE_1_ | Y_o1_ (S·s^n^) | 0.012×10^-4^ | 30.20×10^-4^ | | 4.83×10^-4^ | 34.8×10^-4^ | 39.8×10^-4^ |
|  | N_1_ | 0.870 | 0.890 | 0.563 | | 0.781 | 0.527 |
| R_2_ (Ω·cm^2^) | | - | 503.75 | | 432.49 | 98.525 | 131.71 |
| CPE_2_ | Y_o2_ (S·s^n^) | - | 1007.3×10^-4^ | | 98.1×10^-4^ | 29.5×10^-4^ | 0.646×10^-4^ |
|  | N_2_ | - | 0.307 | | 0.373 | 0.363 | 0.519 |
| W | |  | 7.478×10^-4^ | | 35.7×10^-4^ | 140.5×10^-4^ | 34.5×10^-4^ |
| χ^2^ | | 0.009 | 0.09 | | 0.002 | 0.038 | 0.002 |

Charge carrier density data may be obtained using Mott-Schottky (MS) analysis, which is based on capacitance measurement and is a standard in situ approach for investigating the ionic characteristics of semiconductor polymeric films. The Mott-Schottky plot has a negative slope for all modified electrodes as can be seen in Fig. S4. Polarons or bipolarons function as holes in conventional semiconductors in this scenario.


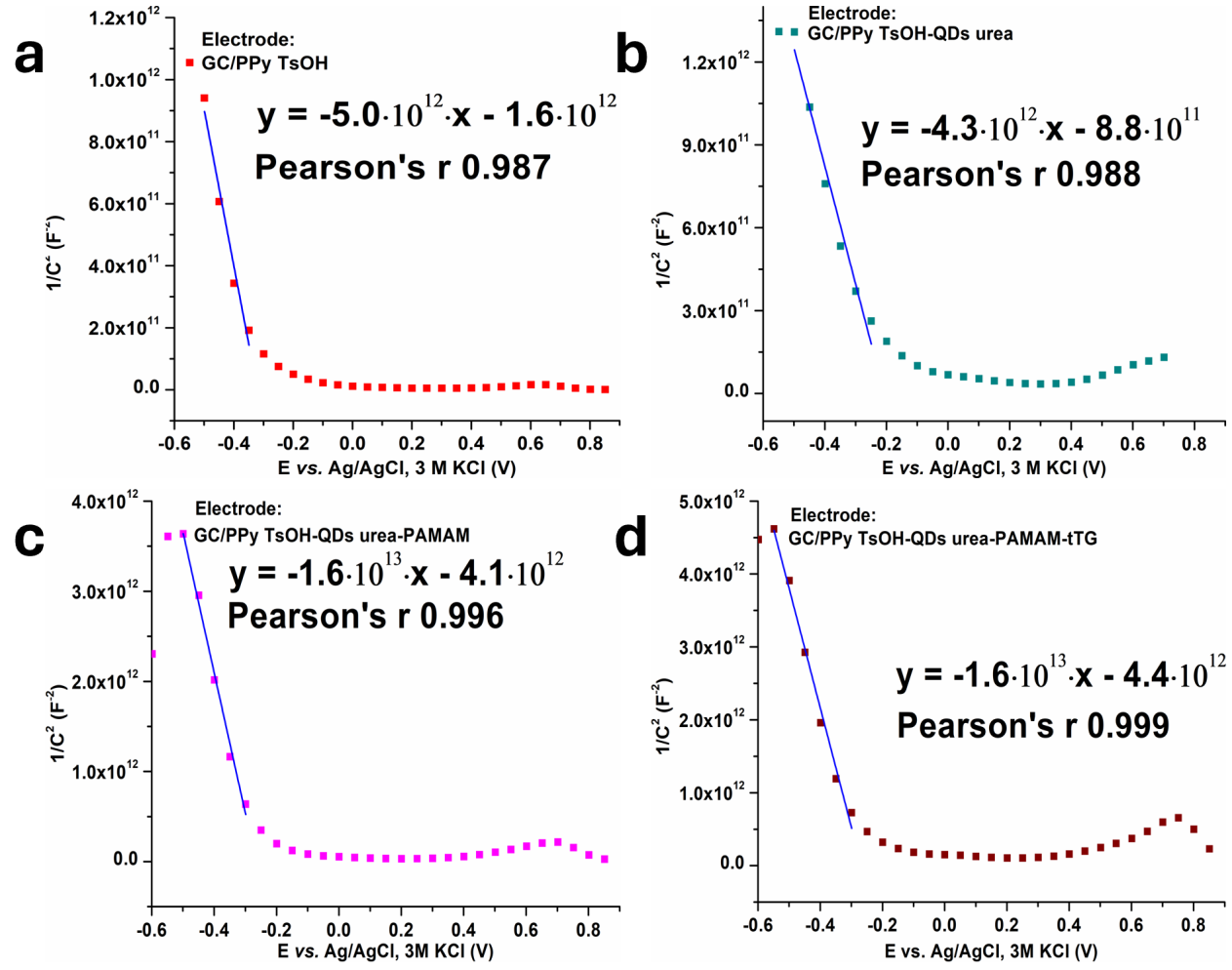


Fig. S5. The Mott–Schottky plots corresponding to the: GC/PPy TsOH (a); GC/PPy TsOH/QDs urea (b), GC/PPy TsOH/QDs urea/PAMAM (c), and GC/PPy TsOH/QDs urea/PAMAM/tTG (d) modified electrodes recorded in 0.1M PBS.

The produced modified electrodes flat band potential (E_FB_) and carrier density (N_D_) may be determined from the MS plots (1/C^2^ as a function of E), according to Equation (1) [29, 30].

$\frac{1}{C^{2}}=\left( \frac{2}{e\varepsilon_{0}\varepsilon_{\mathrm{PPy}}N_{D}} \right)\left[ \left( E-E_{\mathrm{FB}} \right)-\frac{k_{B}T}{e} \right]$ $(1)$

In Eq. 1 above, C stands for the semiconductor's space charge capacitance, e for the elementary charge value (e = 1.60 × 10^−19^ C), ε_0_ for the vacuum permittivity (ε_0_ = 8.85 × 10^−14^ F cm^−1^), ε for the relative permittivity of the polypyrrole semiconductor ($\varepsilon_{\mathrm{PPy}}=10)$, E for the applied voltage, k_B_ for the Boltzmann constant, and T for the temperature [29, 30].

The slope of the linear part of the 1/C^2^ versus applied potential plot may be used to calculate the carrier density of samples using the equation 2 [30]:

$N_{D}=\frac{2}{e\varepsilon_{0}\varepsilon_{\mathrm{PPy}}}{[\frac{d\left( \frac{1}{C^{2}} \right)}{dE}]}^{-1}$ $(2)$

were ${[\frac{d\left( \frac{1}{C^{2}} \right)}{dE}]}^{-1}$ is the slope of the graph. The obtained charge carrier density values are presented in Table S3. The highest density of charge carriers was determined to be for GC/PPy TsOH-QDs urea. Obtained values are similar as order of magnitude with the ones reported in other study [29]. After PAMAM and tTG grafting on the surface, N_D_ drops an order of magnitude and E_FB_ is shifted to more negative values.

| Parameter | Sample | | | | |  |
| --- | --- | --- | --- | --- | --- | --- |
|  | GC/PPy TsOH | | GC/PPy TsOH-QDs urea | | GC/PPy TsOH-QDs urea-PAMAM | GC/PPy TsOH-QDs urea-PAMAM-tTG |
| E_FB_ [V] | -0.320 |  | | -0.207 | -0.265 | - 0.268 |
| N_D_ [cm^-3^] | 2.80 ·10^18^ |  | | 3.305 ·10^18^ | 9.036 ·10^17^ | 8.61 ·10^17^ |

Table S3. Comparison of flatband potentials (E_fb_) and densities of charge carriers (N_D_)

One crucial prerequisite for electrochemical biosensors is the regulated dispersion of [Fe(CN)_6_]^3-/4-^ on surfaces without adsorption [31]. The results depicted in Fig. S5 suggest that that the transmission of electrons between the [Fe(CN)_6_]^3-/4-^ solution and the electrode is more rapid than the concentration gradient-induced migration of electroactive species from the bulk solution to the electrode interface [32].

Modified Randles–Ševčík equation for quasi-reversible processes (Equation 3) can be used to determine the diffusion coefficient (D) of [Fe(CN)_6_]^3-/4-^ from the electrolyte solution to the surface of the unmodified GC electrode. Here, I_p_ is the peak current of the electrode (I_pa_ and I_pc_), n is the number of electrons (1 in this case), A is the geometrical surface area of the GC electrode, ν stands for the scan rate, and C is the concentration in mol/cm^3^ (5·10^-3^ M) and K(Λ, α) is a modified dimensionless parameter for quasi-reversible reactions.

$I_{p}=(2.69\cdot{10}^{5})n^{3/2}AD^{1/2}C\nu^{1/2}K(\Lambda,\alpha$) (3)


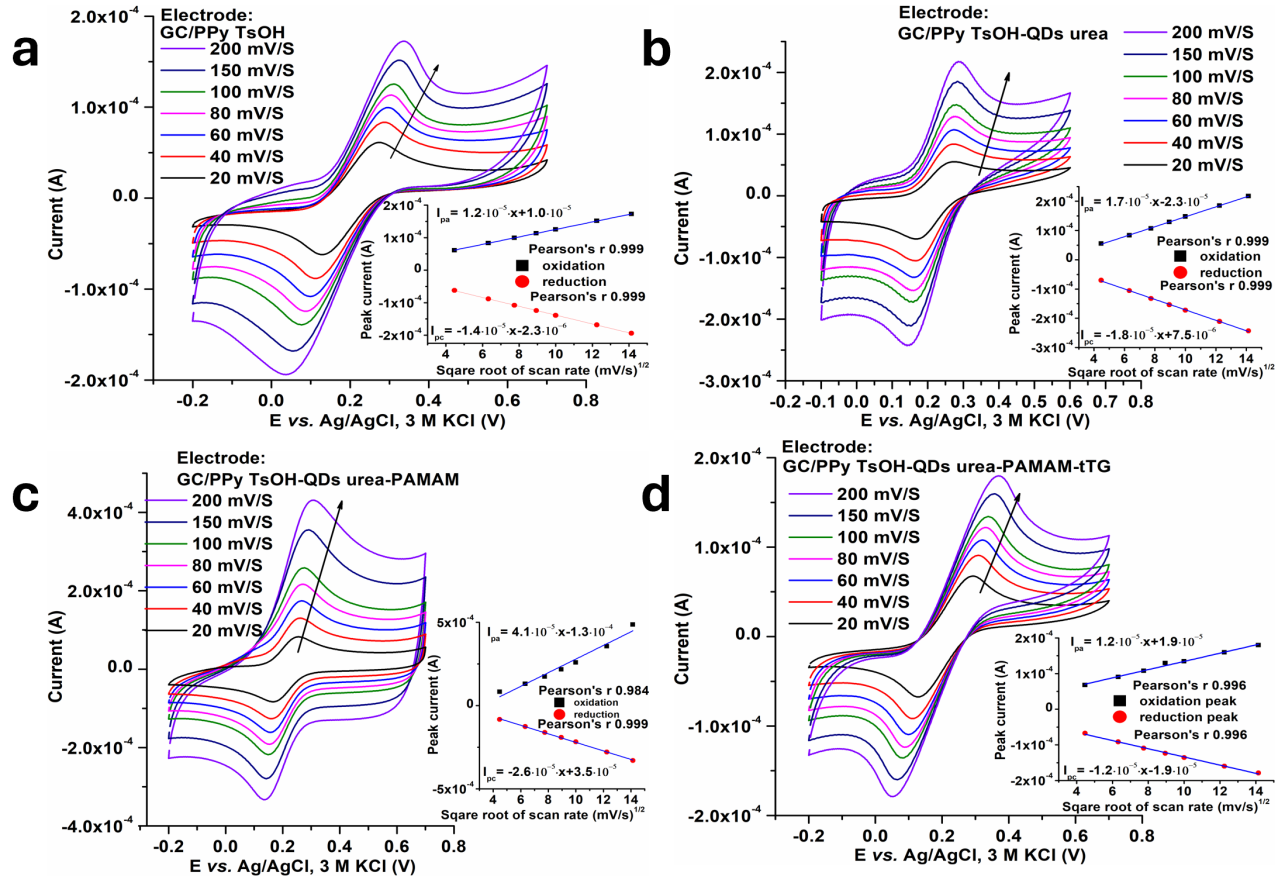


Fig. S6. Cyclic voltammograms of: GC/PPy TsOH (a), GC/PPy TsOH-QDs urea (b), GC/PPy TsOH-QDs urea-PAMAM (c), and GC/PPy TsOH-QDs urea-PAMAM-tTG (d) in 5 mM Fe^2+^/Fe^3+^ PBS solution at different scan rates.

The modified electrode's reaction to albumin, γ-globulin, and creatinine — the three most prevalent blood substances that might obstruct the detection of anti-tTG antibodies can be seen in Fig. S6. For this test, similar electrodes were prepared and incubated for 1 h at room temperature with 30 μL of IgA 0 U/mL anti-tissue antibody standard solution from the Elisa kit, second one in 30 μL of 4 g/dL bovine serum albumin (Sigma Aldrich) solution, the third electrode in 2.5 g/dL γ globulin (Sigma Aldrich) solution, and the fourth electrode in 0.9 mg/mL creatinine (Sigma Aldrich). After rinsing with PBS, DPV measurements were performed in 5 mM Fe^2+^/Fe^3+^ PBS solution.


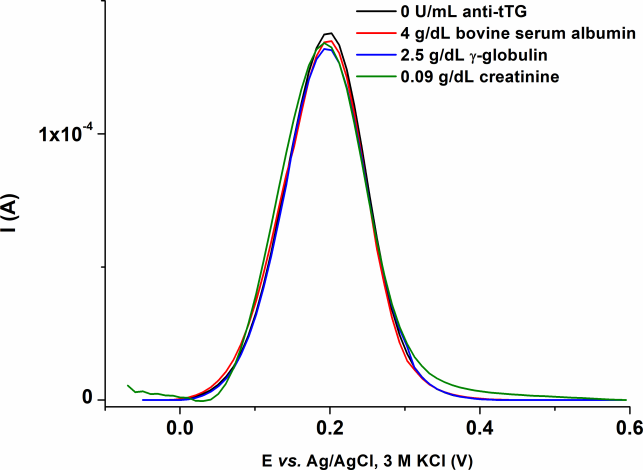


Fig. S7. DPV curves after modified electrode incubation with anti-tissue antibody 0 U/mL, albumin, creatinine, and γ globulin

**References**

1. Jaya Prakash Naik, Prasanta Sutradhar, and Mitali Saha (2017) Molecular scale rapid synthesis of graphene quantum dots (GQDs). Journal of Nanostructure in Chemistry 7: 85-89. 10.1007/s40097-017-0222-9

2. Dan Qu, Min Zheng, Ligong Zhang, Haifeng Zhao, Zhigang Xie, Xiabin Jing, Raid E. Haddad, Hongyou Fan, and Zaicheng Sun (2014) Formation mechanism and optimization of highly luminescent N-doped graphene quantum dots. Sci Rep 4: 5294. <https://doi.org/10.1038/srep05294>

3. Taher Alizadeh and Mahrokh Shokri (2016) A new humidity sensor based upon graphene quantum dots prepared via carbonization of citric acid. Sensors and Actuators B: Chemical 222: 728-734. <https://doi.org/10.1016/j.snb.2015.08.122>

4. Haifang Liu, Zhaohui Li, Yuanqiang Sun, Xin Geng, Yalei Hu, Hongmin Meng, Jia Ge, and Lingbo Qu (2018) Synthesis of Luminescent Carbon Dots with Ultrahigh Quantum Yield and Inherent Folate Receptor-Positive Cancer Cell Targetability. Scientific Reports 8: 1086. 10.1038/s41598-018-19373-3

5. Selin S. Suner, Mehtap Sahiner, Ramesh S. Ayyala, Venkat R. Bhethanabotla, and Nurettin Sahiner (2021) Versatile Fluorescent Carbon Dots from Citric Acid and Cysteine with Antimicrobial, Anti-biofilm, Antioxidant, and AChE Enzyme Inhibition Capabilities. Journal of Fluorescence 31: 1705-1717. 10.1007/s10895-021-02798-x

6. Tingting Yang, Fei Cai, Xiaodan Zhang, and Yuming Huang (2015) Nitrogen and sulfur codoped graphene quantum dots as a new fluorescent probe for Au3+ ions in aqueous media. RSC Advances 5: 107340-107347. 10.1039/C5RA20060A

7. Haiyan Qi, Lixin Qiu, Xiaohong Zhang, Tonghui Yi, Jing Jing, Rokayya Sami, Sitah F. Alanazi, Zahrah Alqahtani, Mahmood D. Aljabri, and Mohammed M. Rahman (2023) Novel N-doped carbon dots derived from citric acid and urea: fluorescent sensing for determination of metronidazole and cytotoxicity studies. RSC Adv 13: 2663-2671. <https://doi.org/10.1039/D2RA07150A>

8. Pichitchai Pimpang, Rattiphorn Sumang, and Supab Choopun (2018) Effect of concentration of citric acid on size and optical properties of fluorescence graphene quantum dots prepared by tuning carbonization degree. Chiang Mai J. Sci 45: 2005.

9. Chang Xia, Xin Hai, Xu-Wei Chen, and Jian-Hua Wang (2017) Simultaneously fabrication of free and solidified N, S-doped graphene quantum dots via a facile solvent-free synthesis route for fluorescent detection. Talanta 168: 269-278. <https://doi.org/10.1016/j.talanta.2017.03.040>

10. Chuanxia Chen, Dan Zhao, Tao Hu, Jian Sun, and Xiurong Yang (2017) Highly fluorescent nitrogen and sulfur co-doped graphene quantum dots for an inner filter effect-based cyanide sensor. Sens Actuators B Chem 241: 779-788. <https://doi.org/10.1016/j.snb.2016.11.010>

11. Mahesh P. More, Pravinkumar H. Lohar, Ashwini G. Patil, Pravin O. Patil, and Prashant K. Deshmukh (2018) Controlled synthesis of blue luminescent graphene quantum dots from carbonized citric acid: Assessment of methodology, stability, and fluorescence in an aqueous environment. Materials Chemistry and Physics 220: 11-22. <https://doi.org/10.1016/j.matchemphys.2018.08.046>

12. Haitao Lin, Jun Huang, and Liyun Ding (2019) Preparation of Carbon Dots with High-Fluorescence Quantum Yield and Their Application in Dopamine Fluorescence Probe and Cellular Imaging. Journal of Nanomaterials 2019: 5037243. 10.1155/2019/5037243

13. Chi-Lin Li, Chih-Ching Huang, Arun Prakash Periasamy, Prathik Roy, Wei-Cheng Wu, Chia-Lun Hsu, and Huan-Tsung Chang (2015) Synthesis of photoluminescent carbon dots for the detection of cobalt ions. RSC Advances 5: 2285-2291. 10.1039/C4RA11704B

14. Hui Liu, Yue Zhang, Jia Hui Liu, Peng Hou, Jun Zhou, and Cheng Zhi Huang (2017) Preparation of nitrogen-doped carbon dots with high quantum yield from Bombyx mori silk for Fe(iii) ions detection. RSC Advances 7: 50584-50590. 10.1039/C7RA10130A

15. Ayşe Merve Şenol, Solomon Bezabeh Kassa, and Yavuz Onganer (2023) A simple fluorescent “Turn off-on” sensor based on P, N-doped graphene quantum dots for Hg^2+^ and Cysteine determination. Sens Actuators A Phys 356: 114362. <https://doi.org/10.1016/j.sna.2023.114362>

16. Niyazbek Ibrayev, Rumiya Dzhanabekova, Evgeniya Seliverstova, and Gulnur Amanzholova (2022) Optical properties of N- and S-doped carbon dots based on citric acid and L-cysteine. Fullerenes, Nanotubes and Carbon Nanostructures 30: 22-26. 10.1080/1536383X.2021.1999933

17. Huifang Wu, Jiahui Jiang, Xiaoting Gu, and Changlun Tong (2017) Nitrogen and sulfur co-doped carbon quantum dots for highly selective and sensitive fluorescent detection of Fe(III) ions and L-cysteine. Microchimica Acta 184: 2291-2298. 10.1007/s00604-017-2201-8

18. Hamidreza Saljoughi, Faeze Khakbaz, and Mohamad Mahani (2020) Synthesis of folic acid conjugated photoluminescent carbon quantum dots with ultrahigh quantum yield for targeted cancer cell fluorescence imaging. Photodiagnosis and Photodynamic Therapy 30: 101687. <https://doi.org/10.1016/j.pdpdt.2020.101687>

19. Wissuta Boonta, Chanon Talodthaisong, Suchinda Sattayaporn, Chiraporn Chaicham, Anusak Chaicham, Somboon Sahasithiwat, Laongdao Kangkaew, and Sirinan Kulchat (2020) The synthesis of nitrogen and sulfur co-doped graphene quantum dots for fluorescence detection of cobalt(ii) ions in water. Materials Chemistry Frontiers 4: 507-516. 10.1039/C9QM00587K

20. C. R. Zamarreño, J. Bravo, J. Goicoechea, I. R. Matias, and F. J. Arregui (2007) Response time enhancement of pH sensing films by means of hydrophilic nanostructured coatings. Sens Actuators B Chem 128: 138-144. <https://doi.org/10.1016/j.snb.2007.05.046>

21. B. Majhy, P. Priyadarshini, and A. K. Sen (2021) Effect of surface energy and roughness on cell adhesion and growth – facile surface modification for enhanced cell culture. RSC Adv 11: 15467-15476. <https://doi.org/10.1039/D1RA02402G>

22. Pi-Guey Su and Wen-Hau Tzou (2012) Low-humidity sensing properties of PAMAM dendrimer and PAMAM–Au nanoparticles measured by a quartz-crystal microbalance. Sens Actuators A Phys 179: 44-49. <https://doi.org/10.1016/j.sna.2012.03.018>

23. Karolina Tokarczyk and Barbara Jachimska (2019) Characterization of G4 PAMAM dendrimer complexes with 5-fluorouracil and their interactions with bovine serum albumin. Colloids Surf A Physicochem Eng Asp 561: 357-363. <https://doi.org/10.1016/j.colsurfa.2018.10.080>

24. Benedict Onyekachi Odii and Peter Coussons (2014) Biological Functionalities of Transglutaminase 2 and the Possibility of Its Compensation by Other Members of the Transglutaminase Family. Sci World J 2014: 714561. <https://doi.org/10.1155/2014/714561>

25. Cristina Dumitriu, Camelia Ungureanu, Simona Popescu, Vlad Tofan, Marian Popescu, and Cristian Pirvu (2015) Ti surface modification with a natural antioxidant and antimicrobial agent. Surf Coat Technol 276: 175-185. <https://doi.org/10.1016/j.surfcoat.2015.06.063>

26. Kasra Vahidi and Yousef Seyed Jalili (2013) Modification of surface energy and electrical and thermal properties of spherical polypyrrole nanoparticles synthesized by CTAB for potential application as a conductive ink. J Theor Appl Phys 7: 42. <https://doi.org/10.1186/2251-7235-7-42>

27. C. P. de Melo, B. B. Neto, L. F. B. Lira, and J. E. G. de Souza (2005) Influence of the nature of the surface of polypyrrole films upon their interaction with volatile organic compounds. Colloids Surf A Physicochem Eng Asp 257-258: 99-103. <https://doi.org/10.1016/j.colsurfa.2004.10.026>

28. Amirhosein Berendjchi, Ramin Khajavi, Ali Akbar Yousefi, and Mohammad Esmail Yazdanshenas (2016) Surface characteristics of coated polyester fabric with reduced graphene oxide and polypyrrole. Applied Surface Science 367: 36-42. <https://doi.org/10.1016/j.apsusc.2016.01.152>

29. Haleh Rasouli, Mir Ghasem Hosseini, Pariya Yardani sefidi, and Solen Kinayyigit (2021) Superior overall water splitting performance in polypyrrole photoelectrode by coupling NrGO and modifying electropolymerization substrate. J Appl Polym Sci 138: 50507. <https://doi.org/10.1002/app.50507>

30. Honey Mittal and Manika Khanuja (2021) Hydrothermal in-situ synthesis of MoSe2-polypyrrole nanocomposite for efficient photocatalytic degradation of dyes under dark and visible light irradiation. Sep Purif Technol 254: 117508. <https://doi.org/10.1016/j.seppur.2020.117508>

31. Jagriti Sethi, Michiel Van Bulck, Ahmed Suhail, Mina Safarzadeh, Ana Perez-Castillo, and Genhua Pan (2020) A label-free biosensor based on graphene and reduced graphene oxide dual-layer for electrochemical determination of beta-amyloid biomarkers. Mikrochim Acta 187: 288. <https://doi.org/10.1007/s00604-020-04267-x>

32. Rocco Cancelliere, David Albano, Benedetta Brugnoli, Katia Buonasera, Gabriella Leo, Andrea Margonelli, and Giuseppina Rea (2021) Electrochemical and morphological layer-by-layer characterization of electrode interfaces during a label-free impedimetric immunosensor build-up: The case of ochratoxin A. Appl Surf Sci 567: 150791. <https://doi.org/10.1016/j.apsusc.2021.150791>
